# Acoustic differences between healthy and depressed people: a cross-situation study

**DOI:** 10.1186/s12888-019-2300-7

**Published:** 2019-10-15

**Authors:** Jingying Wang, Lei Zhang, Tianli Liu, Wei Pan, Bin Hu, Tingshao Zhu

**Affiliations:** 1 Institute of Psychology, Chinese Academy of Sciences, Beijing, China; 2 Department of Computer Science, Virginia Tech, Blacksburg, VA USA; 3 Institute of Population Research, Peking University, Beijing, China; 4 School of Information Science and Engineering, Lanzhou University, Lanzhou, Gansu Province China

**Keywords:** Major depressive disorder, Voice analysis, Acoustic feature, Cross-situation

## Abstract

**Background:**

Abnormalities in vocal expression during a depressed episode have frequently been reported in people with depression, but less is known about if these abnormalities only exist in special situations. In addition, the impacts of irrelevant demographic variables on voice were uncontrolled in previous studies. Therefore, this study compares the vocal differences between depressed and healthy people under various situations with irrelevant variables being regarded as covariates.

**Methods:**

To examine whether the vocal abnormalities in people with depression only exist in special situations, this study compared the vocal differences between healthy people and patients with unipolar depression in 12 situations (speech scenarios). Positive, negative and neutral voice expressions between depressed and healthy people were compared in four tasks. Multiple analysis of covariance (MANCOVA) was used for evaluating the main effects of variable *group* (depressed vs. healthy) on acoustic features. The significances of acoustic features were evaluated by both statistical significance and magnitude of effect size.

**Results:**

The results of multivariate analysis of covariance showed that significant differences between the two groups were observed in all 12 speech scenarios. Although significant acoustic features were not the same in different scenarios, we found that three acoustic features (loudness, MFCC5 and MFCC7) were consistently different between people with and without depression with large effect magnitude.

**Conclusions:**

Vocal differences between depressed and healthy people exist in 12 scenarios. Acoustic features including loudness, MFCC5 and MFCC7 have potentials to be indicators for identifying depression via voice analysis. These findings support that depressed people’s voices include both situation-specific and cross-situational patterns of acoustic features.

## Background

Major depressive disorder (MDD) is one typical mood disorder that can be characterized by a core symptom of consecutive depressed mood. As an approach of emotional expression, voice was found to be linked with neurocognitive dysfunctions for patients with MDD [1]. The voice of a depressed person was summarized as slow, monotonous and disfluent on the basis of previous clinical research, which was quite different from that of healthy people [2]. Empirical studies also revealed that acoustic features have significant relationships with the rating of depression [3–6]. Additionally, they can be utilized for distinguishing depressed people from healthy ones [7–10]. Moreover, the differences of acoustic features between depressed and healthy people have shown relatively high stability over time [11].

It is expected that voice may provide objective clues to assist psychiatrists and clinicians in diagnosing MDD, as well as monitoring response to therapy [12], since it reflects the abnormal changes resulting from MDD and the changes are temporal stable. Nonetheless, a question remains: are the vocal differences in people with depression cross-situational, or can they only be detected in special situations? Answering this question will benefit the design of rational testing environments. If the vocal abnormalities in people with depression only exist in certain special situations, then the testing environment should be arranged to resemble these situations. If the abnormalities are cross-situational, then there are no special requirements on the testing environment. However, few studies [5, 13] have discussed the vocal abnormalities in people with depression in different situations (speech scenarios).

More than one variable has impacts on vocal expression. Therefore, to figure out whether the vocal differences between depressed and healthy people exist in multiple situations, these variables should be regarded as situational conditions when comparing the voices of the two groups.

The first variable is *task*. Different tasks usually have different demands of cognitive function. Cohen [13] compared vocal changes induced by different evocative stimuli like pictures and autobiographical memories. Results revealed that the recall of autobiographical memories could change vocal expression more significantly since it was more personally relevant. Alghowinem et al. [14] found that spontaneous speech caused more vocal variability than reading speech. They argued that acoustic features (e.g., loudness) probably are distinct during spontaneous speech and read speech [14]. In short, different tasks may affect differently on the values of the acoustic features.

The second variable is *emotion*. One study [10] investigated the vocal expression of depressed people in two emotional situations: conceal and non-conceal emotion. Their results indicated that vocal abnormalities in people with depression existed in both conceal and non-conceal conditions. Nevertheless, they did not focus on the vocal differences of depressed people experiencing different emotions. Different emotions have different patterns of vocal expression [15]. In addition, emotion induction (e.g., positive or negative) is a frequently used experimental design for studies of emotional expression of healthy people. In contrast, it was rarely considered in the study of emotional expression in depression. Accordingly, we think that our study, as a cross-situational study, should include emotion as one variable to set speech scenario.

Furthermore, vocal differences also have relationships with some demographic variables such as gender [16]. If these variables have not been excluded when recruiting participants or by being statistically controlled, it is hard to separate out the impact of depression on voice. Therefore, it is necessary to control these influential variables that are significantly discriminative between depressed and healthy people.

In summary, it is important to regard both task and emotion as two situational conditions of speech scenarios to investigate the cross-situational vocal differences between depressed and healthy people with irrelevant variables being regarded as covariates. Consequently, the first aim is to figure out whether the vocal differences between people with and without depression are exist in all situations we considered. To measure the vocal differences, acoustic features of depressed and healthy people were compared under different speech scenarios (situations). If any differences exist in all situations, some acoustic features probably are consistent to identify depression. Therefore, our second aim is to ferret out the potential acoustic features that could be used for identifying depression. If one acoustic feature is significant in all scenarios, it will be considered as an indicator of depression. Based on these aims, we designed various settings of speech scenarios that consisted of different tasks and emotions. We then compared 25 frequently used acoustic features between depressed and healthy people. These acoustic features will be described in the section about feature extraction.

## Method

This experiment was a part of a clinical research project about the potential biological and behavioural indicators of MDD, approved by the ethical board of the Psychology of Institute, Chinese Academy of Science.

### Participants

In this study, we recruited 47 patients who were already diagnosed with MDD from Beijing Anding Hospitals of Capital Medical University, which specializes in mental health. These patients were diagnosed based on DSM-IV criteria [17] by experienced psychologists or psychiatrists. Inclusion criteria included: a) diagnosed as MDD, b) no psychotropic medicines taken within past 2 weeks, c) without mobility difficulties, which could interfere with participation in the study, d) without current or historical DSM-IV diagnosis of any other mental diseases, and e) without current or historical DSM-IV diagnosis of alcohol or drug abuse.

In all, 57 people who matched gender and age with the depressed group and did not have depression (also screened based on DSM-IV by experts) were recruited via local advertisements to form a control group. No participants were diagnosed with other mental diseases.

Table 1 compares the demographic characteristics of depressed people with healthy people. The results denoted that the two groups did not have significant differences in age (t = 1.29, *P* = 0.2) and gender (χ^2^ = 0.04, *P* = 0.85). However, the control group has an obviously higher educational level than the depressive group (χ^2^ = 28.98, *P* < 0.001). Therefore, educational level will be regarded as a covariate in the data analysis.

**Table 1:** Demographic characteristics of the sample

	Depressed (*N* = 47)	Healthy (*N* = 57)
Age (*M* ± *SD*)	34.3 ± 10.3	31.9 ± 8.4
Gender (*n*)		
Female	26	27
Male	21	30
Educational level (*n*)		
Primary school	1	0
Middle school	7	4
High school	5	8
Secondary school	2	1
Junior college	9	1
Bachelor	17	11
Master	6	22
Doctor	0	10

### Speech scenarios

To measure the vocal differences between depressed and healthy people and assess consistency of acoustic features under different situations, we need to design situations first. In our study, we regarded both task and emotion as two situational conditions to form diverse speech scenarios.

The studies about voice analysis of depression designed various tasks (details about the tasks are shown in Additional file 3), including: 1) *interview,* usually originating from interview [3, 7, 8, 18–20]; 2) *natural speech,* in general referring to daily talk or man-machine conversation [10, 21]; 3) *describe or comment picture* [1, 22]; and 4) *reading,* normally conducted by text [5, 6, 9, 10, 23]. In addition, video is a stimulus that is commonly utilized for evoking emotion [24, 25] and could be regarded as a task in our study. Thus, we used videos to form a speech task that asked participants to speak about the video they had watched.

Four tasks were designed based on the aforementioned studies, including “*Video Watching*” (VW), “*Question Answering*” (QA), “*Text Reading*” (TR), and “*Picture Describing*” (PD). Each task involved three emotional materials: positive (happy), negative (sadness) and neutral. All those materials were evaluated for validity before usage. Finally, we conducted a controlled laboratory experiment in 12 speech scenarios (4 tasks × 3 emotions).

After accepting informed consent, participants were seated 1 m away from a 21-in. computer. Information was presented on the computer monitor. The speeches of each participant were received by a professional condenser microphone (Neumann TLM102, Germany) and recorded by a voice recorder (RME Fireface UCX, Germany). The microphone was positioned 50 cm from the right side of the computer. The voice recorder was put at the right side of the computer on the same table. During the experiment, voices of videos, vocal questions and instructions were played via the speaker in the computer. All the recording of vocal questions and instructions were spoken in mandarin.

Participants were asked to complete VW, QA, TR and PD in order (but the order of emotion is random within every task). There are positive, neutral and negative emotional situations in each task, totaling 12 speech scenarios in our experiment.

In task VW, participants first watched a video clip. Then, they were asked to recall the video details based on this instruction “*Which figure or scenario made the strongest impression on you in the last video?*”. For the QA task, participants were asked to orally respond to nine questions (three questions per emotion) one by one (e.g., “*Can you please share with us your most wonderful moment and describe it in detail*?). In the task TR, participants were asked to read three text paragraphs after looking over the text. There are approximately 140 words and one emotion in each text. In the task PD, which included six images, participants were presented with facial expressions or scene images (e.g., a smiling female, a horse sculpture) one by one and asked to think about something associated with the presented image and then to speak about their thoughts. There was a 1-min break between two consecutive tasks.

In each speech scenario, participants were instructed to speak Mandarin as they normally speak. One experimenter controlled the beginning and ending of recording by clicking the button in the software developed by ourselves. Ambient noise was controlled under 50 dB during the experiment. Participants’ speeches were digitally recorded at a sampling frequency of 44.1 kHz and 24-bit sampling using a microphone.

### Feature extraction

The openSMILE software [26] was used to extract acoustic features from the collected voices. In view of the related work, Table 2 shows the 25 acoustic features that were extracted. There are fundamental frequency (F0), loudness, F0 envelope, zero-crossing rate, voicing probability, 12 Mel-frequency cepstrum coefficients (MFCCs) and 8 Line Spectral Pairs (LSP).

**Table 2:** Acoustic features

Name of feature	Explanation
*Loudness*	subjective perception of sound volume
*Fundamental frequency (F0)*	lowest frequency of a periodic waveform
*F0 envelope*	the envelope of the smoothed F0 contour
*Zero-crossing rate (zcr)*	the rate of sign-changes along a signal
*Voicing probability (vp)*	the rate of voicing in one speech
*Mel-frequency cepstrum coefficients (MFCCs)*	vocal tract changes in a certain voice spectral energy
*Line Spectral Pairs (LSPs)*	quantization of linear prediction coefficients (LPC) for transmission over a channel

Some acoustic features have already been investigated in the field of voice analysis of depression. F0 and loudness are the most frequently used features within such studies. Researchers identified a salient correlation between F0 and severity of depression [4, 5, 7, 27]. Loudness has an obvious negative relationship with the rating of depression [6, 21], and the loudness of depressed people is significantly lower than that of healthy people [1, 10]. Furthermore, some studies [28–30] showed that MFCCs can be used to identify depression.

Some acoustic features were rarely utilized in studies about depressed voice, but widely in the field of voice research and surveys. In our study, these features include F0 envelope, zero-crossing rate, voicing probability and Line Spectral Pairs. The F0 envelope is the envelope of the smoothed F0 contour, which is a common feature in affective computing [31]. Zero-crossing rate is the rate of sign-changes along a signal that contributed to detecting emotion from speech [32]. Voicing probability is an indicator of voice quality, and the durations of voiced sounds rely on it [33]. Line Spectral Pairs (LSP) are linear prediction coefficients for filter stability and representational efficiency, which are usually employed in studies of emotion recognition [34].

### Data analysis

It is generally acknowledged that there is a great difference of educational level between depressed and healthy people. Therefore, the impact of educational level needs to be excluded as a covariate when analysing the vocal differences between groups. In this study, multiple analysis of covariance (MANCOVA) was used to compare the differences of acoustic features between groups. All tests are two-tailed, and the level of statistical significance was set at 0.001. The effects of *group* on 25 acoustic features were analysed by the main effect of MANCOVA. Wilks’ Lambda F, *p*-value and partial square of Eta (η_p_^2^) [35] were reported in the analyses of main effect. When relevant, we reported the main effect of *group* on each acoustic feature and used η_p_^2^ to provide insight into the magnitude of group differences. For η_p_^2^, 0.01, 0.06, and 0.14 were considered small, moderate and large effect sizes, respectively [36]. We only regarded the acoustic features with large effect sizes as significant features, because “p < 0.001” was used as the evaluation criterion of significance in this study. The reason for setting this strict criterion (“*p* < 0.001″) is that multiple hypothesis testing was applied in this study and the impact of it should be controlled. The *p*-value of the significant features with large effect sizes (η_p_^2^ ≥ 0.14) was found are all less than 0.001, so the criterion of *p* value was set at 0.001. This criterion is stricter than the criterion calculated by Bonferroni correction. Based on the formula of Bonferroni correction (adjusted p = p / n, n means the number of independent hypotheses which tested in a set of data), the adjusted p-value = 0.05 / 25 = 0.002 (there are 12 dependent multiple testing produced from 12 sets of vocal data. In each testing, there are 25 features conduct to 25 hypotheses).

## Results

Multivariate analyses of covariance (MANCOVA) was calculated to test for main effects of group in each scenario, amounting to 12 separate MANCOVAs. As shown in Table 3, the main effects of *group* were salient in all scenarios, and its effect sizes were all large (to η_p_^2^, 0.14 was considered large). Conversely, the main effects of educational level were not significant in 10 scenarios, except for negative VW and neutral QA. Although there were significant changes on some acoustic features, it indicated the negligible influence on features. In negative VW, educational level had significant impacts on four acoustic features loudness (η_p_^2^ = 0.05), MFCC6 (η_p_^2^ = 0.05), MFCC11 (η_p_^2^ = 0.06) and F0 (η_p_^2^ = 0.06). In neutral QA, educational level has significant influences on 3 acoustic features: loudness (η_p_^2^ = 0.05), MFCC6 (η_p_^2^ = 0.08) and F0 (η_p_^2^ = 0.09).

**Table 3:** The main effect of group in each scenario

Scenario ^a^	Group			Educational Level		
	Wilks’ Lamda (λ)	*P* value	η_p_^2^	Wilks’ Lamda (λ)	P value	η_p_^2^
VW- pos	4.556	.000	.603	1.177	.289	.282
VW- neu	5.894	.000	.666	1.168	.297	.283
VW- neg	4.839	.000	.620	1.683	.045	.362
QA- pos	5.007	.000	.625	1.337	.168	.308
QA- neu	4.659	.000	.608	2.111	.007	.413
QA- neg	5.468	.000	.646	1.579	.068	.345
TR- pos	5.185	.000	.637	1.428	.122	.325
TR- neu	5.369	.000	.645	1.526	.084	.340
TR- neg	5.568	.000	.650	1.559	.073	.342
PD- pos	5.238	.000	.636	0.993	.487	.249
PD- neu	5.427	.000	.644	1.179	.287	.282
PD- neg	4.491	.000	.600	1.387	.141	.316

^a^*VW* video watching, *QA* question answering, *TR* text reading, *PD* picture describing, *pos* positive, *neu* neutral, *neg* negative

To evaluate the voice characteristics of depressed people, the 25 acoustic features of depressed and healthy people were compared by checking their statistical significances. The differences of 25 acoustic features between depressed and healthy people in three types of emotions in four tasks are shown in Tables 4, 5 and 6, respectively. Statistical significances of acoustic features were assessed by computing their effect size values, η_p_^2^, which are also presented in Tables 4, 5 and 6 as well. For η_p_^2^, 0.01, 0.06, and 0.14 were considered small, moderate, and large effect sizes, respectively [36]. Only acoustic features with large effect sizes were considered significant features.

**Table 4:** Positive emotion: the different acoustic features between depressed and healthy people under different tasks

	Video Watching				Question Answering				Text Reading				Picture Describing			
	healthy	depressed	F	η_p_^2^	healthy	depressed	F	η_p_^2^	healthy	depressed	F	η_p_^2^	healthy	depressed	F	η_p_^2^
loudness	0.38 ± 0.17	0.16 ± 0.16	34.07^***^	**.26**	0.38 ± 0.16	0.17 ± 0.17	30.92^***^	.**24**	0.48 ± 0.2	0.23 ± 0.23	24.49^***^	**.20**	0.35 ± 0.16	0.16 ± 0.16	24.61^***^	.**20**
*mfcc1*	−0.32 ± 4.18	0.58 ± 3.81	2.92	.03	0.08 ± 3.34	0.79 ± 3.32	1.30	.01	2.67 ± 3.26	2.69 ± 3.1	0.00	.00	− 0.83 ± 3.76	0.59 ± 3.19	5.44^*^	.05
*mfcc2*	7.81 ± 3.66	8.63 ± 2.70	1.93	.02	8.07 ± 2.93	8.68 ± 2.71	2.07	.02	5.39 ± 4.13	8.35 ± 4.57	13.87^***^	.12	8.29 ± 2.81	9.36 ± 3.01	3.06	.03
*mfcc3*	6.19 ± 4.83	3.28 ± 3.40	9.31^**^	.09	6.98 ± 4.46	3.17 ± 3.35	18.87^***^	.**16**	4.82 ± 5.53	0.34 ± 4.6	14.17^***^	.13	7.27 ± 4.69	3.89 ± 3.73	12.77^**^	.11
*mfcc4*	5.90 ± 4.23	3.94 ± 4.22	6.57^*^	.06	5.04 ± 4.41	3.13 ± 4.25	3.23	.03	0.91 ± 6.13	−0.61 ± 6.71	0.24	.00	6.2 ± 3.95	3.78 ± 4.59	7.56^**^	.07
mfcc5	3.23 ± 6.12	−3.88 ± 6.79	27.60^***^	**.22**	1.67 ± 5.25	−4.93 ± 6.3	26.56^***^	.**21**	− 1.98 ± 6.35	− 10.39 ± 8.97	20.80^***^	**.17**	2.75 ± 5.61	−3.52 ± 5.12	27.07^***^	.**21**
*mfcc6*	3.83 ± 6.88	5.78 ± 6.49	1.10	.01	3.17 ± 5.41	5.95 ± 6.56	3.07	.03	0.34 ± 6.63	5.04 ± 8.16	8.20^**^	.08	3.67 ± 5.34	5.42 ± 6.68	0.68	.01
mfcc7	−0.21 ± 5.32	− 7.25 ± 4.69	47.63^***^	**.33**	− 0.27 ± 5.06	− 7.6 ± 74.11	57.35^***^	.**37**	− 2.12 ± 5.86	− 10.77 ± 4.51	55.24^***^	**.36**	0.19 ± 4.96	− 7.33 ± 3.72	64.00^***^	.**39**
*mfcc8*	2.17 ± 5.51	1.90 ± 4.47	0.18	.00	0.76 ± 5.43	1.87 ± 4.59	1.18	.01	0.44 ± 7.11	1.53 ± 5.63	1.36	.01	1.85 ± 4.92	1.42 ± 4.09	0.43	.00
*mfcc9*	0.33 ± 4.37	2.37 ± 4.15	4.51^*^	.04	− 0.36 ± 5.41	1.67 ± 3.93	3.86	.04	−1.59 ± 6.55	1.01 ± 5.46	6.99^**^	.07	0.39 ± 4.79	2.23 ± 3.57	2.83	.03
*mfcc10*	1.58 ± 5.48	1.29 ± 5.16	0.09	.00	0.83 ± 6.04	0.34 ± 4.99	0.23	.00	−1.42 ± 7.99	− 4.13 ± 7.04	2.21	.02	1.3 ± 5.85	1.01 ± 4.9	0.09	.00
*mfcc11*	− 0.75 ± 5.08	− 0.56 ± 4.20	0.07	.00	− 0.73 ± 4.51	− 0.84 ± 4.14	0.17	.00	− 2.87 ± 5.23	− 3.13 ± 4.71	0.00	.00	−0.64 ± 4.03	−0.01 ± 3.85	0.16	.00
*mfcc12*	−2.17 ± 3.67	− 1.05 ± 2.84	1.10	.01	−1.61 ± 3.56	−1.54 ± 3.05	0.02	.00	−3.02 ± 3.98	−3.18 ± 3.25	0.52	.01	−2.21 ± 3.65	−1.22 ± 2.46	0.58	.01
*lsp0*	0.2 ± 0.04	0.21 ± 0.04	0.08	.00	0.2 ± 0.03	0.2 ± 0.03	0.54	.01	0.19 ± 0.03	0.2 ± 0.02	0.46	.00	0.21 ± 0.04	0.2 ± 0.03	0.02	.00
*lsp1*	0.62 ± 0.06	0.62 ± 0.07	0.36	.00	0.61 ± 0.05	0.61 ± 0.06	0.02	.00	0.55 ± 0.04	0.56 ± 0.05	4.42^*^	.04	0.63 ± 0.06	0.62 ± 0.06	1.14	.01
*lsp2*	0.97 ± 0.07	0.98 ± 0.06	0.38	.00	0.97 ± 0.06	0.98 ± 0.06	1.68	.02	0.92 ± 0.07	0.95 ± 0.06	5.72^*^	.05	0.98 ± 0.06	0.98 ± 0.05	0.00	.00
*lsp3*	1.33 ± 0.07	1.3 ± 0.09	4.70^*^	.05	1.33 ± 0.07	1.29 ± 0.09	3.42	.03	1.28 ± 0.07	1.24 ± 0.08	3.53	.03	1.34 ± 0.07	1.3 ± 0.08	7.39^**^	.07
*lsp4*	1.66 ± 0.08	1.61 ± 0.1	8.55^**^	.08	1.66 ± 0.07	1.6 ± 0.1	10.60^**^	.10	1.62 ± 0.07	1.54 ± 0.1	16.49^***^	.14	1.67 ± 0.07	1.61 ± 0.09	13.44^***^	.12
*lsp5*	1.99 ± 0.07	1.96 ± 0.11	3.24	.03	1.99 ± 0.06	1.94 ± 0.11	4.38^*^	.04	1.95 ± 0.07	1.89 ± 0.11	7.36^**^	.07	2.0 ± 0.06	1.95 ± 0.1	5.50^*^	.05
*lsp6*	2.36 ± 0.07	2.31 ± 0.11	5.18^*^	.05	2.36 ± 0.06	2.3 ± 0.11	9.27^**^	.09	2.33 ± 0.08	2.23 ± 0.13	15.00^***^	.13	2.37 ± 0.06	2.31 ± 0.1	11.37^**^	.10
*lsp7*	2.72 ± 0.04	2.7 ± 0.05	3.13	.03	2.72 ± 0.04	2.69 ± 0.05	6.98^*^	.07	2.7 ± 0.05	2.65 ± 0.07	16.99^***^	**.15**	2.72 ± 0.04	2.7 ± 0.05	7.71^**^	.07
*zcr*	0.03 ± 0.01	0.03 ± 0.01	1.77	.02	0.03 ± 0.01	0.03 ± 0.01	7.95^**^	.07	0.03 ± 0.01	0.04 ± 0.01	13.76^***^	.12	0.03 ± 0.01	0.03 ± 0.01	3.53	.03
*vp*	0.55 ± 0.08	0.51 ± 0.06	8.43^**^	.08	0.56 ± 0.06	0.51 ± 0.05	17.95^***^	.**15**	0.59 ± 0.07	0.57 ± 0.07	7.63^**^	.07	0.55 ± 0.07	0.51 ± 0.05	7.66^**^	.07
*F0*	126.5 ± 54.73	89.62 ± 41.16	10.48^**^	.10	128.32 ± 41.95	90.72 ± 36.79	20.13^***^	**.17**	140.33 ± 39.77	109.61 ± 37.3	18.58^***^	**.16**	124.69 ± 48.05	89.01 ± 37.79	11.59^**^	.10
*F0env*	299.33 ± 38.45	279.74 ± 48.49	4.74^*^	.05	296.98 ± 36.47	274.64 ± 9.57	5.57^*^	.05	266.53 ± 42.48	230.66 ± 43.66	12.78^***^	.11	298.89 ± 37.26	271.72 ± 45.88	9.99^**^	.09

^*^, *p* < 0.05; ^**^, *p* < 0.01; ^***^, *p* < 0.001; In the column of η_p_^2^, we use bold for representing the features have large effect sizes. the upright features represent the features which are significant across all tasks

**Table 5:** Neutral emotion: the different acoustic features between depressed and healthy people under different tasks

	Video Watching				Question Answering				Text Reading				Picture describing			
	healthy	depressed	F	η_p_^2^	healthy	depressed	F	η_p_^2^	healthy	depressed	F	η_p_^2^	healthy	depressed	F	η_p_^2^
loudness	0.37 ± 0.17	0.17 ± 0.17	27.22^***^	.**22**	0.38 ± 0.16	0.17 ± 0.17	26.13^***^	.**21**	0.49 ± 0.21	0.24 ± 0.24	21.20^***^	.**18**	0.34 ± 0.13	0.17 ± 0.17	22.20^***^	.**18**
*mfcc1*	0.06 ± 3.79	0.97 ± 3.63	1.86	.02	0.09 ± 3.45	0.57 ± 3.63	0.92	.01	1.16 ± 3.24	1.63 ± 2.86	0.83	.01	−0.46 ± 3.16	1.02 ± 3.22	7.16^**^	.07
*mfcc2*	8.62 ± 2.96	9.22 ± 3	0.79	.01	8.75 ± 2.73	9.45 ± 2.83	1.08	.01	8.37 ± 3.44	10.93 ± 4.02	11.05^***^	.10	8.35 ± 2.5	8.99 ± 2.25	1.75	.02
*mfcc3*	7.33 ± 4.78	3.8 ± 3.39	15.82^***^	.14	8.09 ± 4.57	3.34 ± 3.49	29.18^***^	.**23**	7.52 ± 5.94	2.46 ± 4.6	21.24^***^	.**18**	7.32 ± 3.83	4.08 ± 3.08	17.81^***^	.**15**
*mfcc4*	6.29 ± 4.65	3.84 ± 4.24	8.64^**^	.08	5.09 ± 4.32	2.98 ± 4.46	5.83^*^	.06	−0.46 ± 6.69	− 2.1 ± 6.39	0.87	.01	6.21 ± 3.53	3.93 ± 3.96	7.44^**^	.07
mfcc5	2.35 ± 6.43	−4.5 ± 6.77	24.71^***^	.**20**	1.77 ± 5.49	−5.83 ± 7.31	31.73^***^	.**24**	−3.6 ± 5.9	− 12.42 ± 8.17	28.81^***^	.**23**	3.32 ± 5.21	− 3.2 ± 4.83	34.84^***^	.**26**
*mfcc6*	4.09 ± 6.64	6.16 ± 6.28	0.83	.01	3.54 ± 5.64	5.5 ± 6.7	0.63	.01	0.34 ± 6.62	5.66 ± 7.66	10.12^**^	.09	4.23 ± 4.42	5.55 ± 6.14	0.24	.00
mfcc7	− 0.21 ± 6.08	− 7.21 ± 4.63	40.80^***^	.**29**	−1 ± 5.28	− 8.08 ± 4.45	49.00^***^	.**33**	−3.81 ± 5.68	− 11.79 ± 4.65	51.12^***^	.**34**	− 0.31 ± 4.3	− 7.06 ± 3.59	64.20^***^	.**39**
*mfcc8*	1.85 ± 5.25	1.97 ± 4.73	0.01	.00	0.35 ± 5.82	1.73 ± 4.65	1.86	.02	−2.78 ± 7.51	− 1.11 ± 5.86	2.25	.02	1.96 ± 3.86	1.85 ± 3.26	0.14	.00
*mfcc9*	0.16 ± 5.01	2.66 ± 3.95	5.91^*^	.06	− 0.59 ± 5.51	1.67 ± 4.05	3.78	.04	−3.27 ± 5.9	− 0.37 ± 4.72	6.10^*^	.06	0.57 ± 4.18	2.4 ± 3.19	3.62	.04
*mfcc10*	1.9 ± 5.7	0.59 ± 5.51	2.01	.02	1.44 ± 5.59	0.06 ± 4.63	3.57	.03	−0.44 ± 6.94	− 2.64 ± 6.22	2.19	.02	1.95 ± 5.04	1.47 ± 4.11	0.41	.00
*mfcc11*	− 0.08 ± 4.46	−0.8 ± 4.46	1.75	.02	− 0.49 ± 4.59	−0.54 ± 3.86	0.34	.00	−1.55 ± 6.06	− 2.2 ± 5.16	0.20	.00	−0.51 ± 3.43	0.19 ± 3.38	0.28	.00
*mfcc12*	−2.2 ± 3.68	−1.1 ± 3.2	0.95	.01	− 1.83 ± 3.75	−1.75 ± 3.16	0.11	.00	−3.23 ± 4.41	−3.47 ± 3.21	0.80	.01	−2 ± 2.8	−1.08 ± 2.11	1.10	.01
*lsp0*	0.19 ± 0.04	0.2 ± 0.04	0.77	.01	0.2 ± 0.03	0.21 ± 0.04	1.68	.02	0.2 ± 0.03	0.2 ± 0.02	0.06	.00	0.2 ± 0.03	0.2 ± 0.03	0.33	.00
*lsp1*	0.62 ± 0.05	0.62 ± 0.07	0.05	.00	0.61 ± 0.05	0.61 ± 0.06	0.71	.01	0.56 ± 0.04	0.57 ± 0.04	2.22	.02	0.63 ± 0.05	0.61 ± 0.05	3.94	.04
*lsp2*	0.98 ± 0.06	0.99 ± 0.06	0.33	.00	0.97 ± 0.06	0.98 ± 0.05	0.22	.00	0.91 ± 0.07	0.95 ± 0.05	8.00^**^	.08	0.99 ± 0.05	0.98 ± 0.05	1.13	.01
*lsp3*	1.34 ± 0.07	1.3 ± 0.09	5.06^*^	.05	1.34 ± 0.07	1.28 ± 0.09	9.67^**^	.09	1.27 ± 0.07	1.23 ± 0.08	2.58	.03	1.35 ± 0.06	1.3 ± 0.07	13.91^***^	.12
*lsp4*	1.67 ± 0.07	1.61 ± 0.1	10.99^***^	.10	1.66 ± 0.07	1.59 ± 0.11	15.73^***^	.14	1.6 ± 0.08	1.52 ± 0.1	14.48^***^	.13	1.68 ± 0.06	1.61 ± 0.08	18.67^***^	.**16**
*lsp5*	2.0 ± 0.07	1.95 ± 0.11	5.46^*^	.05	2.0 ± 0.06	1.94 ± 0.12	7.88^**^	.07	1.94 ± 0.07	1.88 ± 0.1	7.76^**^	.07	2.01 ± 0.05	1.96 ± 0.1	9.47^**^	.09
*lsp6*	2.37 ± 0.06	2.31 ± 0.11	10.24^**^	.09	2.37 ± 0.06	2.29 ± 0.12	14.31^***^	.13	2.32 ± 0.07	2.22 ± 0.12	17.25^***^	.**15**	2.38 ± 0.05	2.32 ± 0.09	15.02^***^	.13
*lsp7*	2.72 ± 0.04	2.7 ± 0.05	5.91^*^	.06	2.72 ± 0.04	2.69 ± 0.06	11.33^***^	.10	2.7 ± 0.05	2.64 ± 0.07	19.24^***^	.**16**	2.73 ± 0.03	2.7 ± 0.04	11.75^***^	.11
*zcr*	0.03 ± 0.01	0.03 ± 0.01	6.30^*^	.06	0.03 ± 0.01	0.03 ± 0.01	15.26^***^	.13	0.03 ± 0.01	0.04 ± 0.01	15.01^***^	.13	0.03 ± 0.01	0.03 ± 0.01	0.81	.01
*vp*	0.56 ± 0.07	0.52 ± 0.06	8.06^**^	.08	0.56 ± 0.06	0.52 ± 0.05	9.81^**^	.09	0.6 ± 0.06	0.57 ± 0.06	8.54^**^	.08	0.54 ± 0.06	0.51 ± 0.05	8.18^**^	.08
*F0*	131.82 ± 57.54	90.43 ± 38.27	13.79^***^	.12	128.75 ± 46.52	93.65 ± 38.37	12.83^***^	.11	144.87 ± 36.71	111.44 ± 35.54	23.83^***^	.**20**	120.79 ± 42.4	84.84 ± 36.85	13.67^***^	.12
*F0env*	297.16 ± 40.91	271.51 ± 45.86	8.58^**^	.08	296.22 ± 40.37	269.71 ± 50.15	9.07^**^	.08	267.89 ± 40.63	266.53 ± 42.48	16.56^***^	.14	305.99 ± 30.86	272.83 ± 41.98	20.20^***^	.**17**

^*^, *p* < 0.05; ^**^, *p* < 0.01; ^***^, *p* < 0.001; In the column of η_p_^2^, we use bold for representing the features have large effect sizes. the upright features represent the features which are significant across all tasks

**Table 6:** Negative emotion: the different acoustic features between depressed and healthy people under different tasks

	Video Watching				Question Answering				Text Reading				Picture describing			
	healthy	depressed	F	η_p_^2^	healthy	depressed	F	η_p_^2^	healthy	depressed	F	η_p_^2^	healthy	depressed	F	η_p_^2^
loudness	0.35 ± 0.14	0.16 ± 0.16	28.47^***^	.**22**	0.35 ± 0.15	0.16 ± 0.16	28.55^***^	.**22**	0.48 ± 0.21	0.23 ± 0.22	25.57^***^	.**21**	0.35 ± 0.14	0.17 ± 0.17	23.58^***^	.**19**
*mfcc1*	−0.29 ± 3.61	0.74 ± 3.81	2.03	.02	−0.37 ± 3.22	0.7 ± 3.91	2.26	.02	1.23 ± 3.16	1.16 ± 2.83	0.00	.00	−0.23 ± 3.67	1.14 ± 3.22	4.38^*^	.04
*mfcc2*	8.07 ± 3.17	9.07 ± 3.18	2.17	.02	8.39 ± 2.86	8.88 ± 3.27	0.66	.01	8.05 ± 3.53	10.88 ± 4.15	13.65^***^	.12	8.22 ± 2.5	8.82 ± 2.63	1.26	.01
*mfcc3*	6.9 ± 4.83	3 ± 3.72	16.13^***^	.14	7.42 ± 4.21	2.9 ± 3.47	29.55^***^	.**23**	7.49 ± 5.7	2.65 ± 4.19	18.44^***^	**.16**	6.68 ± 4.66	3.44 ± 3.56	11.25^***^	.10
*mfcc4*	6.39 ± 4.13	4.16 ± 4.05	7.16^**^	.07	6.0 ± 4.05	3.26 ± 4.43	9.97^**^	.09	0.76 ± 6.35	−1.04 ± 6.44	0.62	.01	5.68 ± 3.87	3.52 ± 4.59	5.48^*^	.05
mfcc5	3.15 ± 5.4	− 4.4 ± 6.49	35.88^***^	.**27**	2.93 ± 5.57	− 4.37 ± 6.94	28.87^***^	.**23**	− 2.61 ± 6.2	−11.26 ± 7.75	27.26^***^	.**22**	3.1 ± 4.95	−3.51 ± 5.17	36.73^***^	.**27**
*mfcc6*	3.85 ± 6.21	5.72 ± 6.88	0.54	.01	4.33 ± 5.68	6.0 ± 6.51	0.47	.00	0.72 ± 6.87	5.48 ± 8.15	7.73^**^	.07	4.2 ± 5.39	6.26 ± 6.73	1.05	.01
mfcc7	0.02 ± 5.5	− 7.46 ± 4.46	52.57^***^	.**35**	− 0.02 ± 4.93	−7.51 ± 4.51	58.30^***^	.**37**	− 3.02 ± 5.37	− 11.55 ± 4.97	55.80^***^	.**36**	−0.04 ± 5.08	−6.95 ± 3.66	54.37^***^	.**35**
*mfcc8*	1.86 ± 5.25	1.72 ± 4.71	0.05	.00	1.22 ± 5.04	1.8 ± 4.62	0.30	.00	− 2.48 ± 8.24	−0.51 ± 6.01	3.09	.03	2.26 ± 4.43	1.61 ± 3.88	0.70	.01
*mfcc9*	0.28 ± 4.86	2.56 ± 4.08	5.09^*^	.05	0.35 ± 5.33	1.96 ± 3.78	2.18	.02	− 2.81 ± 6.25	− 0.01 ± 5	6.28^*^	.06	0.64 ± 4.78	2.42 ± 3.71	3.05	.03
*mfcc10*	1.62 ± 5.62	0.42 ± 5.03	1.55	.02	2.21 ± 5.32	0.52 ± 4.39	4.73^*^	.05	− 1.1 ± 7.37	− 3.51 ± 6.39	1.86	.02	1.68 ± 5.47	1.21 ± 4.97	0.29	.00
*mfcc11*	−1.17 ± 4.27	−1.29 ± 3.83	0.88	.01	−0.42 ± 4.25	−0.35 ± 4	0.28	.00	−2.73 ± 5.8	−2.63 ± 5.04	0.13	.00	−0.56 ± 3.93	−0.14 ± 4	0.02	.00
*mfcc12*	− 2.03 ± 3.5	− 1.04 ± 3.41	1.12	.01	−1.4 ± 3.53	−1.06 ± 2.83	0.09	.00	−3.31 ± 3.96	−3.72 ± 3.47	0.75	.01	−1.98 ± 3.3	−1.26 ± 2.44	0.35	.00
*lsp0*	0.2 ± 0.03	0.2 ± 0.04	0.45	.00	0.2 ± 0.03	0.21 ± 0.04	0.87	.01	0.2 ± 0.03	0.2 ± 0.02	1.05	.01	0.2 ± 0.04	0.2 ± 0.03	0.09	.00
*lsp1*	0.63 ± 0.05	0.62 ± 0.06	0.47	.00	0.63 ± 0.05	0.61 ± 0.07	1.16	.01	0.57 ± 0.04	0.58 ± 0.04	5.51^*^	.05	0.63 ± 0.05	0.61 ± 0.06	1.83	.02
*lsp2*	0.98 ± 0.06	0.99 ± 0.05	0.42	.00	0.98 ± 0.06	0.98 ± 0.06	0.00	.00	0.92 ± 0.07	0.95 ± 0.05	9.53^**^	.09	0.99 ± 0.06	0.98 ± 0.05	0.10	.00
*lsp3*	1.34 ± 0.07	1.3 ± 0.09	7.05^**^	.07	1.34 ± 0.06	1.3 ± 0.09	8.82^**^	.08	1.28 ± 0.07	1.24 ± 0.08	2.34	.02	1.35 ± 0.06	1.3 ± 0.08	10.17^**^	.09
*lsp4*	1.67 ± 0.07	1.61 ± 0.1	11.87^***^	.11	1.68 ± 0.06	1.6 ± 0.11	15.23^***^	.13	1.61 ± 0.08	1.53 ± 0.1	13.03^***^	.12	1.68 ± 0.06	1.61 ± 0.09	15.65^***^	.14
*lsp5*	2.0 ± 0.06	1.95 ± 0.11	5.91^*^	.06	2.0 ± 0.05	1.95 ± 0.12	7.66^**^	.07	1.94 ± 0.07	1.88 ± 0.11	6.16^*^	.06	2.0 ± 0.06	1.95 ± 0.1	7.73^**^	.07
*lsp6*	2.37 ± 0.06	2.3 ± 0.11	9.87^**^	.09	2.38 ± 0.05	2.31 ± 0.11	13.83^***^	.12	2.32 ± 0.07	2.23 ± 0.13	16.43^***^	.14	2.38 ± 0.06	2.31 ± 0.1	13.30^***^	.12
*lsp7*	2.72 ± 0.04	2.7 ± 0.06	7.80^**^	.07	2.73 ± 0.03	2.7 ± 0.05	11.11^***^	.10	2.7 ± 0.05	2.65 ± 0.07	15.37^***^	.13	2.73 ± 0.04	2.7 ± 0.05	7.50^**^	.07
*zcr*	0.03 ± 0.01	0.03 ± 0.01	7.68^**^	.07	0.03 ± 0.01	0.03 ± 0.01	9.07^**^	.08	0.03 ± 0.01	0.04 ± 0.01	14.70^***^	.13	0.03 ± 0.01	0.03 ± 0.01	1.85	.02
*vp*	0.55 ± 0.07	0.52 ± 0.05	5.67^*^	.05	0.54 ± 0.06	0.51 ± 0.05	7.34^**^	.07	0.6 ± 0.07	0.58 ± 0.07	9.10^**^	.08	0.55 ± 0.06	0.51 ± 0.05	8.00^**^	.07
*F0*	125.48 ± 51.38	92.61 ± 41.25	8.01^**^	.07	121.39 ± 45.68	87.43 ± 38.27	11.46^***^	.10	147.96 ± 38.73	114.42 ± 37.68	21.22^***^	.**18**	122.53 ± 44.32	88.1 ± 37.42	12.32^***^	.11
*F0env*	298.82 ± 39.73	275.57 ± 47.45	6.47^*^	.06	302.75 ± 36.76	277.74 ± 49.99	7.53^**^	.07	271.56 ± 40.91	235.74 ± 42.61	14.26^***^	.13	304.12 ± 35.79	272.66 ± 46.23	12.46^***^	.11

^*^, *p* < 0.05; ^**^, *p* < 0.01; ^***^, *p* < 0.001; In the column of η_p_^2^, we use bold for representing the features have large effect sizes. the upright features represent the features which are significant across all tasks

It can easily be observed (see Tables 4, 5 and 6) that the significant acoustic features were distinguished in different speech scenarios. There were 5.75 significant acoustic features on average under neutral emotional scenarios. By contrast, the mean number of significant features was 4.5 in both positive and negative emotional scenarios. The comparison of the number of significant acoustic features among different tasks indicated that TR had the largest mean significant features (6.7), compared with VW (3.7), QA (5) and PD (4.3).

**Fig. 1:**
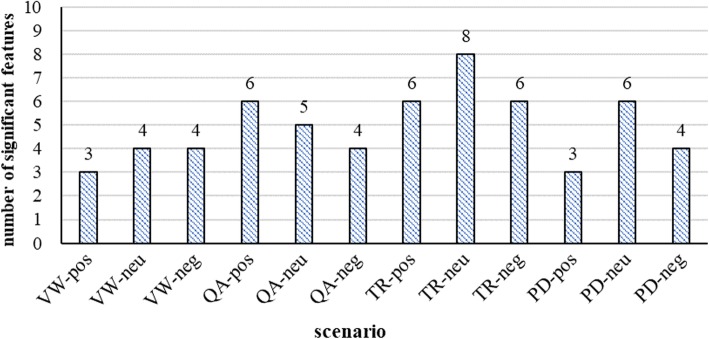
The number of significant acoustic features in each scenario (Task: VW, video watching; QA, question answering; TR, text reading; PD, picture describing. Emotion: pos, positive; neu, neutral; neg, negative)

The number of significant acoustic features was calculated in each scenario. There were approximately five significant acoustic features on average. As shown in Fig.1, each scenario had acoustic features ranging from 3 to 8 that were statistically discriminative between depressed and healthy people.

Tables 4, 5 and 6 show that the values of η_p_^2^ revealed evident vocal differences in loudness, MFCC5 and MFCC7 between the groups, no matter which emotion or task the scenario was involved. The means of the three features of healthy people were all consistent and higher than those of depressed people in every scenario. That is to say, there were not only significant differences in acoustic features between groups, but the magnitude of these differences was large enough to be considered meaningful.

In addition, acoustic features F0 and MFCC3 had large effect sizes in some scenarios and moderate effect sizes in other scenarios.

## Discussion

This study sought to help determine whether vocal differences between depressed and healthy people exist across various speech scenarios. We set up 3 (emotion) × 4 (task) speech scenarios to examine 25 acoustic features of 47 depressed people versus 57 healthy people. Notable strengths of the present study are, first, exclusion of the impact of covariate educational level; and second, use of statistical test and effect sizes to evaluate both statistical significance and effect magnitude. The results of MANCOVA in 12 speech scenarios showed 12 valid main effects of group with large effect sizes. There were five significant acoustic features on average between depressed and healthy people under 12 scenarios. Moreover, some acoustic features of depressed people were found to be consistently higher than those of healthy people.

One key finding in this study is that vocal differences between depressed and healthy people exist in all speech scenarios. The results of MANCOVA reported 12 valid main effects of group with large effect sizes, which means the vocal abnormalities in depressed people exist in various emotional or cognitive scenarios. Compared with the previous studies that usually compared among different tasks [5, 10, 14], we set up more multiple speech scenarios that included more diverse tasks (represented different cognitive demands) and added another influential variable emotion, while excluding the covariates. Therefore, our study provides more reliable evidence of the cross-situational vocal abnormalities in depressed people.

Although our study suggested that the voice abnormalities in depressed people exist in various situations, there were different significant discriminative acoustic features (the quantity range from 3 to 8) between people with and without depression in 12 different scenarios. This finding revealed that depressed voices include both cross-situational existence of abnormal acoustic features and situation-specific patterns of acoustic features.

Another key finding is that the acoustic features loudness, MFCC5 and MFCC7 are consistent (Additional file 4). They were statistically significant with large effect sizes across 12 speech scenarios. Loudness is defined as sound volume. In our study, the Loudness of healthy people was obviously louder than that of depressed people. This aligns with clinical observation [2] and a previous study [14] that supported that depression is associated with a decrease in loudness. MFCCs are coefficients of Mel-frequency cepstrum (MFC), which is a representation of the short-term power spectrum of a sound. MFCCs reflected vocal tract changes [37]. Taguchi et al. [30] found a distinguishable difference of MFCC2 between depressed and healthy people. In contrast, we have not found a difference of MFCC2, but found other differences in MFCC5 and MFCC7*.* The two coefficients of healthy people were visibly higher than those of depressed people. We speculate that these differences suggest depressed people have less vocal tract changes compared with healthy people, due to the symptom named psychomotor retardation that leads to a tight vocal tract. There is also a brain evidence to explain the differences of MFCCs between the two groups. The study of Keedwell [38] stated that the neural responses in inferior frontal gyrus (IFG) has a salient negative relationship with anhedonia in major depressive disorder. Furthermore, the left posterior IFG is a part of the motor syllable programs involved in phonological processing [39, 40]. That is to say, the decrease of MFCCs in depressed people possibly is an outcome derived from the reduction of neural responses in IFG, which results in less speech motor. The result that lower MFCCs in depressed people in our study is in accord with it, because lower MFCCs represents less vocal tract changes (equals to less vocal tract movements). Additionally, for those cross-situational significant features loudness, MFCC5 and MFCC7, we found that educational level has a mild influence on loudness in both negative VW and neutral QA, but not influence on MFCC5 and MFCC7. According to this result, we believe that MFCCs is a steadier type of acoustic feature to reflect the vocal difference between depressed and healthy people.

In addition, we found depressed F0 and MFCC3 were pronounced and significantly lower than in healthy people in some speech scenarios. It was consistent with several previous studies that demonstrated that F0 has a dramatic negative relationship with depression severity [41] and increased after positive treatment [5]. It was reported that F0 had a positive relationship with the overall muscle tension of the speaker [42], which possibly symbolized a weak voice in depressed people. A lower MFCC3 in depressed people again indicated that depressed people have less vocal tract changes than healthy people because of their tight vocal tracts. Additionally, as a high-risk factor of depression, suicidal behaviours have significant relationships with some acoustic features [43]. F0 and MFCCs are distinctly different between suicidal and non-suicidal groups.

An additional interesting finding is that the acoustic features loudness, F0, MFCC3, MFCC5 and MFCC7 were smaller in people with depression than in healthy people in all scenarios. These vocal differences indicate that the depressed voice is untoned, low-pitched and weak. This finding provides powerful evidences for supporting the theory of emotion context insensitivity [44] which claimed that the emotional response of depression is generally flatter than normal emotional reaction, regardless of emotional type.

Gender difference also need to be mentioned. The result (Additional file 1 and Additional file 2) shows that the differences of MFCC3 between depressed and healthy people are significant only in males. This finding accords with a previous study [45] which found that MFCC features are help for gender detection.

Several limitations of this study should be mentioned. First, the small sample size limited the generalizability of our findings. Second, educational level of health group is high in this study because we adopted convenience sampling in an area surrounded by many research institutes. It is another limitation which might impact the generalizability of this study. In general, MDD patients have lower education degrees than their health controls [46, 47]. Furthermore, the impact of educational level was controlled as a covariate during data analysis. Therefore, the influence of educational difference should be reasonably controlled. Even so, we should be cautious about the generalizability of this result while considering the indirect correlation between education and depression. That is, low education degree probably leads to low income, while low income is a risk factor of depression [48]. In addition, our sample focuses on major depressive disorder. Thus, the conclusion of this study should not simply be generalized to other kinds of depression.

For future research, the experimental paradigm of this study should be repeated in a larger sample with a stricter sampling strategy. Besides, these are three themes could be considered for the further investigation. One theme is about the vocal differences among different depression severities which might have different quantities or types of abnormal acoustic features. One theme is to compare the vocal differences between different time by adding follow-up data. For example, comparing the vocal differences between the time before and after treatment for evaluating the response to therapy. Future studies also should investigate whether the vocal features are steady across languages. Although Pitch (F0) was found remarkably similar across languages and cultures [49], other features have not been proved significant across languages. So the language we used might limit the generalizability to other languages, considering Mandarin is very different from other common-used languages like English, Germany.

## Conclusion

In our study, the voices of 47 depressed people were compared with the voices of 57 healthy people throughout 12 speech scenarios. Our results pointed out that the vocal differences between depressed and healthy people follow both cross-situational and situation-specific patterns, and loudness, MFCC5 and MFCC7 are effective indicators that could be utilized for identifying depression. These findings supported that there are no special requirements on testing environment while identifying depression via voice analysis, but it is better to utilize loudness, MFCC5 and MFCC7 for modelling.

## Supplementary information

**Additional file 1: Table S1.** Positive emotion: the different acoustic features between depressed and healthy people under different tasks (female). **Table S2.** Neutral emotion: the different acoustic features between depressed and healthy people under different tasks (female). **Table S3.** Negative emotion: the different acoustic features between depressed and healthy people under different tasks (female).

**Additional file 2: Table S1.** Positive emotion: the different acoustic features between depressed and healthy people under different tasks (male). **Table S2.** Neutral emotion: the different acoustic features between depressed and healthy people under different tasks (male). **Table S3.** Negative emotion: the different acoustic features between depressed and healthy people under different tasks (male).

**Additional file 3.** Stimuli in the tasks.

**Additional file 4.** Box-whisker plots of loudness, MFCC5, and MFCC7 in each emotion.

## Data Availability

Data of this study are not publicity available as being a part of a broader project, which data are still analyzing, but are available from the corresponding author on reasonable request.

## Abbreviations

F0: Fundamental frequency
LSP: Line spectral pair
MANCOVA: Multiple analysis of covariance
MDD: Major depressive disorder
MFCC: Mel-frequency cepstrum coefficient
PD: Picture describing
QA: Question answering
TR: Text reading
vp: Voicing probability
VW: Video watching
zcr: Zero-crossing rate
