# Supplementary material for: Acoustic differences between healthy and depressed people: a cross-situation study
Source: BMC Psychiatry. 2019 Oct 15;19:300. doi: 10.1186/s12888-019-2300-7 (PMC6794822; doi:10.1186/s12888-019-2300-7)
Supplement: Supplementary file 1 — Additional file 1: Table S1. Positive emotion: the different acoustic features between depressed and healthy people under different tasks (female). Table S2. Neutral emotion: the different acoustic features between depressed and healthy people under different tasks (female). Table S3. Negative emotion: the different acoustic features between depressed and healthy people under different tasks (female). [file 12888_2019_2300_MOESM1_ESM.docx]

**Table 1 Positive emotion: the different acoustic features between depressed and healthy people under different tasks (female)**

|  | ***Video Watching*** | | | | ***Question Answering*** | | | | ***Text Reading*** | | | | ***Picture Describing*** | | | |
| --- | --- | --- | --- | --- | --- | --- | --- | --- | --- | --- | --- | --- | --- | --- | --- | --- |
|  | ***healthy*** | ***depressed*** | F | η_p_^2^ | ***healthy*** | ***depressed*** | F | η_p_^2^ | ***healthy*** | ***depressed*** | F | η_p_^2^ | ***healthy*** | ***depressed*** | F | η_p_^2^ |
| ***loudness*** | 0.36 ± 0.15 | 0.17 ± 0.17 | 9.16^***^ | **.27** | 0.34 ± 0.12 | 0.18 ± 0.18 | 7.39^**^ | .23 | 0.44 ± 0.15 | 0.26 ± 0.25 | 4.74^*^ | .16 | 0.32 ± 0.11 | 0.18 ± 0.18 | 5.45^**^ | .18 |
| ***mfcc1*** | -1.77 ± 3.24 | -0.25 ± 4.05 | 2.25 | .08 | -1.59 ± 3.23 | -0.19 ± 3.32 | 3.03 | .11 | 1.21 ± 2.85 | 1.62 ± 3.03 | 4.53^*^ | .15 | -2.65 ± 3.37 | -0.14 ± 3.08 | 5.55^**^ | .18 |
| ***mfcc2*** | 7.04 ± 3.90 | 8.83 ± 2.45 | 2.38 | .09 | 7.16 ± 2.66 | 8.90 ± 2.88 | 2.82 | .10 | 3.97 ± 3.57 | 8.04 ± 4.70 | 6.52^**^ | .21 | 7.67 ± 3.00 | 9.38 ± 2.91 | 3.25^*^ | .12 |
| ***mfcc3*** | 4.01 ± 4.14 | 2.49 ± 3.44 | 1.15 | .04 | 4.49 ± 3.68 | 2.41 ± 2.82 | 2.60 | .09 | 1.53 ± 4.56 | -1.14 ± 3.39 | 3.20^*^ | .11 | 4.88 ± 4.03 | 2.97 ± 3.54 | 1.85 | .07 |
| ***mfcc4*** | 4.90 ± 4.49 | 3.12 ± 4.68 | 1.85 | .07 | 3.65 ± 3.70 | 2.25 ± 4.52 | 0.95 | .04 | -0.84 ± 5.50 | -2.74 ± 6.93 | 0.62 | .02 | 5.10 ± 3.72 | 2.22 ± 4.51 | 3.90^*^ | .14 |
| ***mfcc5*** | 2.08 ± 5.41 | -3.97 ± 7.45 | 6.47^**^ | .21 | 0.87 ± 4.27 | -5.37 ± 6.61 | 8.27^***^ | **.25** | -3.54 ± 5.79 | -12.22 ± 9.09 | 9.82^***^ | **.28** | 2.16 ± 4.56 | -3.84 ± 5.36 | 9.53^***^ | **.28** |
| ***mfcc6*** | 1.65 ± 6.57 | 4.13 ± 6.14 | 2.24 | .08 | 1.94 ± 4.61 | 4.55 ± 6.95 | 2.39 | .09 | -1.77 ± 6.70 | 3.17 ± 8.80 | 4.11^*^ | .14 | 2.76 ± 4.91 | 3.74 ± 6.89 | 0.79 | .03 |
| ***mfcc7*** | 0.14 ± 5.02 | -6.47 ± 4.52 | 15.74^***^ | **.39** | 0.08 ± 4.60 | -7.17 ± 3.44 | 21.62^***^ | **.46** | -1.51 ± 5.80 | -11.34 ± 4.35 | 24.10^***^ | **.49** | 0.55 ± 4.87 | -6.85 ± 3.08 | 22.11^***^ | **.47** |
| ***mfcc8*** | 0.21 ± 4.87 | 1.33 ± 4.31 | 1.73 | .07 | -1.61 ± 4.54 | 1.33 ± 4.82 | 4.64^*^ | .16 | -3.24 ± 5.96 | 0.99 ± 4.86 | 4.24^*^ | .15 | -0.12 ± 3.30 | 0.55 ± 3.91 | 1.74 | .07 |
| ***mfcc9*** | -0.44 ± 4.73 | 1.42 ± 3.76 | 1.89 | .07 | -2.17 ± 5.28 | 0.47 ± 4.00 | 2.68 | .10 | -4.73 ± 5.99 | -1.33 ± 5.35 | 2.33 | .09 | -0.75 ± 4.90 | 1.26 ± 3.45 | 3.42^*^ | .12 |
| ***mfcc10*** | -1.38 ± 5.33 | -0.22 ± 5.71 | 5.96^**^ | .19 | -2.42 ± 6.26 | -1.33 ± 5.36 | 5.66^**^ | .19 | -6.62 ± 7.97 | -7.53 ± 6.97 | 0.97 | .04 | -1.88 ± 6.10 | -0.64 ± 4.93 | 4.30^*^ | .15 |
| ***mfcc11*** | -2.92 ± 5.49 | -1.61 ± 4.43 | 4.07^*^ | .14 | -3.09 ± 3.59 | -1.71 ± 4.48 | 3.65^*^ | .13 | -5.69 ± 4.74 | -4.99 ± 4.24 | 0.38 | .02 | -2.44 ± 4.43 | -1.19 ± 4.05 | 3.79^*^ | .13 |
| ***mfcc12*** | -1.34 ± 2.81 | -0.52 ± 2.78 | 1.37 | .05 | -0.75 ± 3.76 | -1.14 ± 2.59 | 0.10 | .00 | -1.03 ± 3.36 | -2.93 ± 3.74 | 1.92 | .07 | -1.11 ± 2.99 | -0.89 ± 2.56 | 1.28 | .05 |
| ***lsp0*** | 0.21 ± 0.04 | 0.21 ± 0.05 | 1.12 | .04 | 0.21 ± 0.03 | 0.21 ± 0.04 | 2.23 | .08 | 0.20 ± 0.02 | 0.20 ± 0.02 | 3.00 | .11 | 0.22 ± 0.03 | 0.21 ± 0.03 | 1.61 | .06 |
| ***lsp1*** | 0.63 ± 0.06 | 0.63 ± 0.07 | 0.09 | .00 | 0.62 ± 0.05 | 0.62 ± 0.06 | 0.03 | .00 | 0.55 ± 0.04 | 0.57 ± 0.05 | 3.39^*^ | .12 | 0.64 ± 0.06 | 0.63 ± 0.06 | 0.78 | .03 |
| ***lsp2*** | 0.98 ± 0.07 | 0.99 ± 0.05 | 0.48 | .02 | 0.98 ± 0.05 | 0.99 ± 0.05 | 0.54 | .02 | 0.93 ± 0.06 | 0.96 ± 0.05 | 1.64 | .06 | 0.99 ± 0.05 | 0.99 ± 0.04 | 0.11 | .00 |
| ***lsp3*** | 1.33 ± 0.08 | 1.31 ± 0.09 | 1.10 | .04 | 1.33 ± 0.06 | 1.30 ± 0.09 | 0.81 | .03 | 1.28 ± 0.07 | 1.24 ± 0.09 | 2.36 | .09 | 1.35 ± 0.07 | 1.31 ± 0.08 | 1.67 | .06 |
| ***lsp4*** | 1.67 ± 0.07 | 1.62 ± 0.11 | 2.48 | .09 | 1.67 ± 0.06 | 1.61 ± 0.11 | 3.21^*^ | .11 | 1.63 ± 0.07 | 1.54 ± 0.10 | 6.46^*^ | .21 | 1.68 ± 0.06 | 1.62 ± 0.09 | 4.02^*^ | .14 |
| ***lsp5*** | 1.99 ± 0.07 | 1.97 ± 0.11 | 0.76 | .03 | 1.99 ± 0.05 | 1.96 ± 0.11 | 0.74 | .03 | 1.94 ± 0.07 | 1.90 ± 0.11 | 1.81 | .07 | 2.00 ± 0.06 | 1.97 ± 0.11 | 1.00 | .04 |
| ***lsp6*** | 2.36 ± 0.07 | 2.32 ± 0.12 | 1.49 | .06 | 2.36 ± 0.06 | 2.31 ± 0.12 | 2.10 | .08 | 2.32 ± 0.08 | 2.23 ± 0.14 | 4.07^*^ | .14 | 2.37 ± 0.06 | 2.32 ± 0.12 | 2.47 | .09 |
| ***lsp7*** | 2.72 ± 0.04 | 2.70 ± 0.05 | 1.76 | .07 | 2.72 ± 0.04 | 2.70 ± 0.05 | 2.48 | .09 | 2.70 ± 0.05 | 2.64 ± 0.08 | 5.96^**^ | .19 | 2.72 ± 0.04 | 2.70 ± 0.05 | 3.00 | .11 |
| ***zcr*** | 0.03 ± 0.01 | 0.04 ± 0.01 | 1.59 | .06 | 0.03 ± 0.01 | 0.04 ± 0.01 | 4.16^*^ | .14 | 0.03 ± 0.01 | 0.04 ± 0.01 | 7.60^**^ | .23 | 0.03 ± 0.01 | 0.04 ± 0.01 | 2.00 | .07 |
| ***vp*** | 0.58 ± 0.07 | 0.53 ± 0.06 | 11.03^***^ | .31 | 0.59 ± 0.05 | 0.54 ± 0.06 | 12.57^***^ | **.33** | 0.64 ± 0.06 | 0.61 ± 0.06 | 2.72 | .10 | 0.57 ± 0.07 | 0.53 ± 0.05 | 10.26^***^ | **.29** |
| ***F0*** | 136.18 ± 50.29 | 103.17 ± 43.13 | 11.51^***^ | **.32** | 143.1 ± 38.7 | 104.18 ± 39.96 | 13.96^***^ | **.36** | 158.82 ± 28.82 | 127.75 ± 34.4 | 10.44^***^ | **.29** | 132.49 ± 45.23 | 99.5 ± 40.49 | 9.99^***^ | **.29** |
| ***F0env*** | 305.67 ± 25.23 | 291 ± 42.77 | 1.90 | .07 | 303.86 ± 25.17 | 282.15 ± 43.98 | 2.42 | .09 | 276.19 ± 35.79 | 238.65 ± 33.88 | 7.97^**^ | .24 | 308.26 ± 23.22 | 281.1 ± 42.22 | 4.36^*^ | .15 |

Note: ^*^, p < 0.05; ^**^, p < 0.01; ^***^, p < 0.001; In the column of η_p_^2^, we use bold for representing the features have large effect sizes.

**Table 2 Neutral emotion: the different acoustic features between depressed and healthy people under different tasks (female)**

|  | ***Video Watching*** | | | | ***Question Answering*** | | | | ***Text Reading*** | | | | ***Picture Describing*** | | | |
| --- | --- | --- | --- | --- | --- | --- | --- | --- | --- | --- | --- | --- | --- | --- | --- | --- |
|  | ***healthy*** | ***depressed*** | F | η_p_^2^ | ***healthy*** | ***depressed*** | F | η_p_^2^ | ***healthy*** | ***depressed*** | F | η_p_^2^ | ***healthy*** | ***depressed*** | F | η_p_^2^ |
| ***loudness*** | 0.33 ± 0.11 | 0.19 ± 0.18 | 5.99^**^ | .19 | 0.33 ± 0.12 | 0.18 ± 0.17 | 6.41^**^ | .20 | 0.45 ± 0.16 | 0.27 ± 0.26 | 4.81^*^ | .16 | 0.31 ± 0.10 | 0.19 ± 0.19 | 4.60^*^ | .16 |
| ***mfcc1*** | -1.80 ± 3.68 | 0.11 ± 3.69 | 3.14 | .11 | -1.80 ± 3.03 | -0.31 ± 3.91 | 2.61 | .10 | -0.25 ± 2.58 | 0.70 ± 3.09 | 3.26^*^ | .12 | -2.06 ± 3.06 | 0.36 ± 3.39 | 4.14^*^ | .14 |
| ***mfcc2*** | 8.12 ± 3.13 | 9.56 ± 3.36 | 3.63^*^ | .13 | 8.56 ± 2.58 | 9.72 ± 3.05 | 1.59 | .06 | 7.86 ± 3.16 | 10.76 ± 4.03 | 4.43^*^ | .15 | 7.61 ± 2.25 | 9.05 ± 2.19 | 3.50^*^ | .12 |
| ***mfcc3*** | 5.41 ± 4.47 | 2.81 ± 3.05 | 3.81^*^ | .13 | 5.57 ± 3.43 | 2.24 ± 3.00 | 6.97^**^ | .22 | 3.62 ± 3.97 | 0.16 ± 2.88 | 6.55^**^ | .21 | 5.42 ± 3.02 | 3.61 ± 2.86 | 2.46 | .09 |
| ***mfcc4*** | 5.66 ± 4.97 | 2.90 ± 4.12 | 4.84^*^ | .16 | 3.78 ± 3.88 | 1.56 ± 4.49 | 3.59^*^ | .13 | -3.06 ± 6.37 | -4.91 ± 5.83 | 1.39 | .05 | 5.15 ± 3.31 | 2.89 ± 4.32 | 2.64 | .10 |
| ***mfcc5*** | 1.62 ± 6.07 | -4.92 ± 7.39 | 6.71^**^ | .21 | 1.57 ± 4.42 | -6.35 ± 7.82 | 10.90^***^ | **.30** | -3.76 ± 4.92 | -13.65 ± 8.61 | 13.92^***^ | **.36** | 2.98 ± 4.32 | -3.36 ± 5.30 | 11.41^***^ | **.31** |
| ***mfcc6*** | 2.74 ± 6.37 | 4.41 ± 6.51 | 2.78 | .10 | 2.66 ± 5.11 | 4.16 ± 7.06 | 2.31 | .08 | -1.34 ± 6.47 | 3.83 ± 8.14 | 6.06^**^ | .20 | 3.15 ± 3.75 | 4.35 ± 6.72 | 0.94 | .04 |
| ***mfcc7*** | 0.84 ± 5.78 | -6.93 ± 3.76 | 20.62^***^ | **.45** | -0.51 ± 4.80 | -7.50 ± 3.38 | 21.61^***^ | **.46** | -4.10 ± 5.01 | -12.97 ± 4.09 | 24.76^***^ | **.50** | 0.06 ± 4.33 | -6.58 ± 2.53 | 25.03^***^ | **.50** |
| ***mfcc8*** | 0.46 ± 4.12 | 1.15 ± 4.71 | 2.55 | .09 | -2.43 ± 4.93 | 1.19 ± 4.74 | 7.08^**^ | .22 | -6.71 ± 7.17 | -2.31 ± 5.35 | 3.46^*^ | .12 | 0.31 ± 3.09 | 1.40 ± 3.06 | 2.45 | .09 |
| ***mfcc9*** | -0.66 ± 5.10 | 1.55 ± 3.21 | 3.48^*^ | .12 | -1.95 ± 5.30 | 0.49 ± 4.09 | 5.37^**^ | .18 | -4.75 ± 6.00 | -1.62 ± 4.93 | 2.86 | .10 | -0.94 ± 4.93 | 1.80 ± 3.73 | 5.12^**^ | .17 |
| ***mfcc10*** | -1.02 ± 5.63 | -1.65 ± 5.70 | 6.32^**^ | .20 | -1.47 ± 6.01 | -1.55 ± 4.91 | 9.78^***^ | **.28** | -3.62 ± 7.64 | -5.06 ± 6.36 | 1.61 | .06 | -0.57 ± 5.65 | 0.44 ± 4.61 | 3.37^*^ | .12 |
| ***mfcc11*** | -2.09 ± 4.44 | -2.31 ± 4.10 | 3.92^*^ | .14 | -2.78 ± 3.85 | -1.45 ± 4.10 | 6.02^**^ | .19 | -5.60 ± 5.20 | -4.96 ± 3.81 | 1.44 | .05 | -2.09 ± 3.77 | -0.94 ± 3.40 | 4.44^*^ | .15 |
| ***mfcc12*** | -1.00 ± 3.17 | -0.67 ± 3.30 | 0.80 | .03 | -0.87 ± 3.50 | -1.52 ± 3.02 | 1.27 | .05 | -1.27 ± 3.64 | -3.47 ± 3.91 | 2.21 | .08 | -1.19 ± 2.64 | -0.84 ± 2.01 | 4.41^*^ | .15 |
| ***lsp0*** | 0.21 ± 0.04 | 0.21 ± 0.04 | 2.79 | .10 | 0.21 ± 0.03 | 0.21 ± 0.04 | 2.64 | .10 | 0.21 ± 0.02 | 0.21 ± 0.02 | 2.15 | .08 | 0.21 ± 0.03 | 0.20 ± 0.04 | 1.52 | .06 |
| ***lsp1*** | 0.64 ± 0.05 | 0.63 ± 0.07 | 0.36 | .01 | 0.63 ± 0.05 | 0.62 ± 0.07 | 0.34 | .01 | 0.57 ± 0.03 | 0.58 ± 0.05 | 1.00 | .04 | 0.64 ± 0.04 | 0.62 ± 0.05 | 1.70 | .06 |
| ***lsp2*** | 1.00 ± 0.05 | 1.00 ± 0.05 | 0.57 | .02 | 0.99 ± 0.05 | 0.99 ± 0.05 | 0.11 | .00 | 0.93 ± 0.06 | 0.96 ± 0.05 | 2.08 | .08 | 1.00 ± 0.05 | 0.99 ± 0.04 | 0.53 | .02 |
| ***lsp3*** | 1.35 ± 0.06 | 1.31 ± 0.10 | 2.15 | .08 | 1.34 ± 0.06 | 1.30 ± 0.10 | 2.41 | .09 | 1.27 ± 0.07 | 1.24 ± 0.08 | 1.78 | .07 | 1.36 ± 0.05 | 1.31 ± 0.07 | 3.62^*^ | .13 |
| ***lsp4*** | 1.69 ± 0.06 | 1.62 ± 0.10 | 4.40^*^ | .15 | 1.68 ± 0.06 | 1.61 ± 0.11 | 4.89^*^ | .16 | 1.62 ± 0.07 | 1.54 ± 0.10 | 5.45^**^ | .18 | 1.69 ± 0.05 | 1.63 ± 0.08 | 6.03^**^ | .19 |
| ***lsp5*** | 2.00 ± 0.06 | 1.97 ± 0.12 | 1.59 | .06 | 2.00 ± 0.05 | 1.96 ± 0.12 | 1.66 | .06 | 1.93 ± 0.07 | 1.89 ± 0.11 | 1.60 | .06 | 2.01 ± 0.05 | 1.97 ± 0.10 | 2.03 | .08 |
| ***lsp6*** | 2.37 ± 0.06 | 2.31 ± 0.13 | 2.96 | .11 | 2.37 ± 0.06 | 2.30 ± 0.13 | 3.75^*^ | .13 | 2.31 ± 0.08 | 2.22 ± 0.14 | 4.35^*^ | .15 | 2.38 ± 0.04 | 2.33 ± 0.10 | 3.69^*^ | .13 |
| ***lsp7*** | 2.72 ± 0.04 | 2.70 ± 0.06 | 2.76 | .10 | 2.72 ± 0.04 | 2.69 ± 0.06 | 5.36^**^ | .18 | 2.69 ± 0.05 | 2.63 ± 0.08 | 6.66^**^ | .21 | 2.73 ± 0.03 | 2.71 ± 0.04 | 5.26^**^ | .17 |
| ***zcr*** | 0.03 ± 0.01 | 0.03 ± 0.01 | 4.18^*^ | .14 | 0.03 ± 0.01 | 0.04 ± 0.01 | 7.36^**^ | .23 | 0.04 ± 0.01 | 0.05 ± 0.01 | 7.18^**^ | .22 | 0.03 ± 0.01 | 0.03 ± 0.01 | 2.13 | .08 |
| ***vp*** | 0.58 ± 0.07 | 0.54 ± 0.06 | 6.71^**^ | .21 | 0.58 ± 0.06 | 0.54 ± 0.06 | 15.66^***^ | **.39** | 0.64 ± 0.05 | 0.61 ± 0.06 | 5.83^**^ | .19 | 0.55 ± 0.06 | 0.52 ± 0.05 | 9.85^***^ | **.28** |
| ***F0*** | 141.83 ± 62.92 | 103.64 ± 41.85 | 5.76^**^ | .19 | 143.7 ± 47.4 | 107.46 ± 42.92 | 13.72^***^ | **.35** | 162.33 ± 26.49 | 127.93 ± 31.37 | 16.18^***^ | **.39** | 122.82 ± 43.18 | 95.33 ± 40.78 | 10.76^***^ | **.30** |
| ***F0env*** | 308.81 ± 29.65 | 278.69 ± 40.89 | 4.85^*^ | .16 | 311.7 ± 25.65 | 281.41 ± 42.14 | 5.02^*^ | .17 | 277.85 ± 28.29 | 239.46 ± 29.53 | 11.77^***^ | **.32** | 314.04 ± 22.05 | 278.04 ± 42.39 | 7.53^**^ | .23 |

Note: ^*^, p < 0.05; ^**^, p < 0.01; ^***^, p < 0.001; In the column of η_p_^2^, we use bold for representing the features have large effect sizes.

**Table 3 Negative emotion: the different acoustic features between depressed and healthy people under different tasks (female)**

|  | ***Video Watching*** | | | | ***Question Answering*** | | | | ***Text Reading*** | | | | ***Picture Describing*** | | | |
| --- | --- | --- | --- | --- | --- | --- | --- | --- | --- | --- | --- | --- | --- | --- | --- | --- |
|  | ***healthy*** | ***depressed*** | F | η_p_^2^ | ***healthy*** | ***depressed*** | F | η_p_^2^ | ***healthy*** | ***depressed*** | F | η_p_^2^ | ***healthy*** | ***depressed*** | F | η_p_^2^ |
| ***loudness*** | 0.32 ± 0.11 | 0.18 ± 0.17 | 6.62^**^ | .21 | 0.32 ± 0.11 | 0.17 ± 0.17 | 6.90^**^ | .22 | 0.46 ± 0.16 | 0.26 ± 0.24 | 6.05^**^ | .20 | 0.32 ± 0.11 | 0.18 ± 0.19 | 5.54^**^ | .18 |
| ***mfcc1*** | -1.52 ± 3.38 | 0.25 ± 4.18 | 2.69 | .10 | -1.65 ± 3.20 | -0.27 ± 4.21 | 1.56 | .06 | -0.31 ± 2.64 | 0.20 ± 2.89 | 2.46 | .09 | -1.85 ± 3.50 | 0.55 ± 3.07 | 5.60^**^ | .18 |
| ***mfcc2*** | 7.34 ± 3.03 | 8.71 ± 3.65 | 4.58^*^ | .16 | 7.99 ± 2.58 | 8.92 ± 3.74 | 0.75 | .03 | 7.04 ± 2.73 | 11.07 ± 4.34 | 8.63^**^ | .26 | 7.61 ± 2.03 | 8.89 ± 2.70 | 3.14 | .11 |
| ***mfcc3*** | 4.54 ± 4.02 | 2.41 ± 3.42 | 2.76 | .10 | 5.41 ± 3.57 | 1.86 ± 3.16 | 7.20^**^ | .22 | 3.71 ± 3.95 | 0.99 ± 2.83 | 5.55^**^ | .18 | 4.54 ± 4.15 | 2.70 ± 3.47 | 1.51 | .06 |
| ***mfcc4*** | 5.31 ± 3.03 | 3.18 ± 3.90 | 3.32^*^ | .12^*^ | 4.96 ± 3.75 | 2.36 ± 4.50 | 4.85^*^ | .16 | -1.96 ± 5.69 | -3.55 ± 6.30 | 0.51 | .02 | 4.32 ± 3.27 | 2.11 ± 4.62 | 2.92 | .10 |
| ***mfcc5*** | 2.55 ± 4.50 | -4.76 ± 6.77 | 11.72^***^ | **.32** | 2.39 ± 4.17 | -4.56 ± 7.61 | 9.44^***^ | **.27** | -3.89 ± 5.33 | -12.68 ± 7.96 | 12.79^***^ | **.34** | 2.68 ± 3.66 | -3.91 ± 5.46 | 13.60^***^ | **.35** |
| ***mfcc6*** | 2.62 ± 5.31 | 4.42 ± 7.69 | 2.67 | .10 | 3.15 ± 4.90 | 4.46 ± 6.80 | 1.69 | .06 | -1.49 ± 6.96 | 3.62 ± 8.88 | 4.87 | .16 | 2.64 ± 4.31 | 4.78 ± 6.71 | 2.44 | .09 |
| ***mfcc7*** | 0.49 ± 5.06 | -7.15 ± 3.56 | 21.54^***^ | **.46** | 0.47 ± 4.37 | -7.30 ± 4.08 | 23.28^***^ | **.48** | -3.88 ± 4.97 | -12.33 ± 4.38 | 21.09^***^ | **.46** | 0.03 ± 5.21 | -6.66 ± 3.05 | 17.53^***^ | **.41** |
| ***mfcc8*** | -0.15 ± 4.29 | 1.18 ± 5.14 | 3.17 | .11 | -0.61 ± 4.57 | 1.05 ± 4.55 | 3.99^*^ | .14 | -6.76 ± 7.59 | -1.60 ± 5.75 | 4.01^*^ | .14 | 0.29 ± 3.26 | 0.88 ± 3.57 | 0.97 | .04 |
| ***mfcc9*** | -0.70 ± 4.40 | 1.69 ± 3.99 | 5.49^**^ | .18 | -0.89 ± 5.10 | 0.79 ± 3.88 | 2.94 | .11 | -4.88 ± 5.99 | -1.56 ± 4.92 | 2.58 | .09 | -0.93 ± 5.02 | 1.32 ± 3.77 | 3.04 | .11 |
| ***mfcc10*** | -1.65 ± 5.41 | -1.78 ± 4.66 | 8.06^**^ | .24 | -0.53 ± 5.79 | -0.72 ± 4.58 | 6.80^**^ | .21 | -5.25 ± 7.61 | -6.6 ± 6.24 | 1.31 | .05 | -1.42 ± 5.65 | -0.40 ± 5.23 | 3.59^*^ | .13 |
| ***mfcc11*** | -2.84 ± 4.41 | -2.70 ± 3.70 | 7.73^**^ | .24 | -2.11 ± 4.31 | -0.96 ± 4.32 | 6.30^**^ | .20 | -6.31 ± 5.04 | -4.94 ± 4.13 | 1.85 | .07 | -2.43 ± 4.12 | -1.57 ± 3.87 | 3.82^*^ | .13 |
| ***mfcc12*** | -1.26 ± 3.43 | -0.04 ± 2.73 | 1.48 | .06 | -0.25 ± 3.50 | -0.74 ± 2.43 | 0.31 | .01 | -1.80 ± 3.76 | -3.49 ± 4.00 | 1.28 | .05 | -0.99 ± 3.04 | -1.15 ± 2.42 | 0.72 | .03 |
| ***lsp0*** | 0.21 ± 0.04 | 0.21 ± 0.04 | 2.52 | .09 | 0.21 ± 0.03 | 0.22 ± 0.05 | 1.14 | .04 | 0.21 ± 0.02 | 0.21 ± 0.02 | 0.80 | .03 | 0.21 ± 0.04 | 0.20 ± 0.03 | 3.26^*^ | .12 |
| ***lsp1*** | 0.64 ± 0.05 | 0.62 ± 0.07 | 1.06 | .04 | 0.64 ± 0.05 | 0.62 ± 0.07 | 0.78 | .03 | 0.57 ± 0.03 | 0.59 ± 0.05 | 2.96 | .11 | 0.64 ± 0.04 | 0.62 ± 0.06 | 0.85 | .03 |
| ***lsp2*** | 1.00 ± 0.05 | 1.00 ± 0.05 | 0.58 | .02 | 0.99 ± 0.05 | 0.99 ± 0.05 | 0.21 | .01 | 0.93 ± 0.06 | 0.97 ± 0.05 | 3.11 | .11 | 0.99 ± 0.05 | 0.99 ± 0.04 | 0.00 | .00 |
| ***lsp3*** | 1.36 ± 0.06 | 1.31 ± 0.09 | 3.78^*^ | .13 | 1.35 ± 0.05 | 1.31 ± 0.09 | 2.81 | .10 | 1.28 ± 0.07 | 1.24 ± 0.09 | 2.45 | .09 | 1.35 ± 0.05 | 1.31 ± 0.08 | 2.40 | .09 |
| ***lsp4*** | 1.69 ± 0.06 | 1.62 ± 0.10 | 5.95^**^ | .19 | 1.69 ± 0.05 | 1.62 ± 0.11 | 5.05^**^ | .17 | 1.62 ± 0.07 | 1.54 ± 0.10 | 5.54^**^ | .18 | 1.68 ± 0.06 | 1.62 ± 0.09 | 4.53^*^ | .15 |
| ***lsp5*** | 2.01 ± 0.05 | 1.97 ± 0.12 | 2.07 | .08 | 2.01 ± 0.04 | 1.97 ± 0.12 | 2.03 | .08 | 1.93 ± 0.06 | 1.90 ± 0.11 | 1.16 | .04 | 2.00 ± 0.05 | 1.97 ± 0.10 | 1.41 | .05 |
| ***lsp6*** | 2.38 ± 0.06 | 2.31 ± 0.13 | 3.47^*^ | .12 | 2.38 ± 0.04 | 2.31 ± 0.12 | 4.09^*^ | .14 | 2.31 ± 0.07 | 2.22 ± 0.14 | 4.13^*^ | .14 | 2.37 ± 0.06 | 2.32 ± 0.11 | 2.55 | .09 |
| ***lsp7*** | 2.72 ± 0.04 | 2.70 ± 0.06 | 4.51^*^ | .15 | 2.73 ± 0.03 | 2.70 ± 0.05 | 6.03^**^ | .19 | 2.69 ± 0.04 | 2.64 ± 0.08 | 5.12^**^ | .17 | 2.72 ± 0.04 | 2.71 ± 0.05 | 2.30 | .08 |
| ***zcr*** | 0.03 ± 0.01 | 0.04 ± 0.01 | 5.62^**^ | .18 | 0.03 ± 0.01 | 0.04 ± 0.01 | 5.12^**^ | .17 | 0.04 ± 0.01 | 0.04 ± 0.01 | 4.27^*^ | .15 | 0.03 ± 0.01 | 0.03 ± 0.01 | 3.28^*^ | .12 |
| ***vp*** | 0.57 ± 0.07 | 0.54 ± 0.05 | 9.76^***^ | **.28** | 0.56 ± 0.06 | 0.53 ± 0.06 | 10.69^***^ | **.30** | 0.65 ± 0.05 | 0.62 ± 0.06 | 4.61^*^ | .16 | 0.57 ± 0.06 | 0.53 ± 0.06 | 11.58^***^ | **.32** |
| ***F0*** | 135.65 ± 51.86 | 106.09 ± 44.48 | 8.93^***^ | **.26** | 130.54 ± 47.67 | 100.45 ± 44.3 | 11.27^***^ | **.31** | 164.44 ± 26.9 | 133.54± 33.31 | 12.07^***^ | **.33** | 131.01 ± 41.67 | 101.65 ± 40.22 | 13.30^***^ | **.35** |
| ***F0env*** | 309.93 ± 32.3 | 280.85 ± 39.58 | 4.36^*^ | .15 | 313.68 ± 24.99 | 285.99 ±45.43 | 4.58^*^ | .16 | 280.89 ± 30.71 | 245.54 ±33.37 | 8.11^**^ | .25 | 310.09 ± 21.18 | 277.35 ± 43.77 | 6.15^**^ | .20 |

Note: ^*^, p < 0.05; ^**^, p < 0.01; ^***^, p < 0.001; In the column of η_p_^2^, we use bold for representing the features have large effect sizes.
