# Supplementary material for: Acoustic differences between healthy and depressed people: a cross-situation study
Source: BMC Psychiatry. 2019 Oct 15;19:300. doi: 10.1186/s12888-019-2300-7 (PMC6794822; doi:10.1186/s12888-019-2300-7)
Supplement: Supplementary file 2 — Additional file 2: Table S1. Positive emotion: the different acoustic features between depressed and healthy people under different tasks (male). Table S2. Neutral emotion: the different acoustic features between depressed and healthy people under different tasks (male). Table S3. Negative emotion: the different acoustic features between depressed and healthy people under different tasks (male). [file 12888_2019_2300_MOESM2_ESM.docx]

**Table 1 Positive emotion: the different acoustic features between depressed and healthy people under different tasks (male)**

|  | ***Video Watching*** | | | | ***Question Answering*** | | | | ***Text Reading*** | | | | | ***Picture Describing*** | | | |
| --- | --- | --- | --- | --- | --- | --- | --- | --- | --- | --- | --- | --- | --- | --- | --- | --- | --- |
|  | ***healthy*** | ***depressed*** | F | η_p_^2^ | ***healthy*** | ***depressed*** | F | η_p_^2^ | | ***healthy*** | ***depressed*** | F | η_p_^2^ | ***healthy*** | ***depressed*** | F | η_p_^2^ |
| ***loudness*** | 0.40 ± 0.19 | 0.14 ± 0.15 | 16.81^***^ | **.41** | 0.41 ± 0.19 | 0.15 ± 0.15 | 17.45^***^ | **.42** | | 0.51 ± 0.23 | 0.20 ± 0.20 | 14.14^***^ | **.37** | 0.39 ± 0.18 | 0.14 ± 0.18 | 18.14^***^ | **.43** |
| ***mfcc1*** | 0.99 ± 4.54 | 1.61 ± 3.29 | 0.38 | .02 | 1.58 ± 2.68 | 2.00 ± 2.97 | 0.14 | .01 | | 3.98 ± 3.08 | 4.00 ± 2.70 | 0.16 | .01 | 0.81 ± 3.35 | 1.49 ± 3.35 | 0.47 | .02 |
| ***mfcc2*** | 8.51 ± 3.35 | 8.38 ± 3.03 | 0.02 | .00 | 8.88 ± 2.97 | 8.41 ± 2.52 | 0.40 | .02 | | 6.67 ± 4.24 | 8.73 ± 4.49 | 1.45 | .06 | 8.85 ± 2.56 | 9.34 ± 2.56 | 0.24 | .01 |
| ***mfcc3*** | 8.15 ± 4.61 | 4.25 ± 3.15 | 5.53^**^ | .19 | 9.22 ± 3.90 | 4.12 ± 3.76 | 11.15^***^ | **.32** | | 7.79 ± 4.61 | 2.17 ± 5.28 | 9.35^***^ | **.28** | 9.42 ± 4.22 | 5.03 ± 4.22 | 7.57^**^ | .24 |
| ***mfcc4*** | 6.79 ± 3.83 | 4.96 ± 3.41 | 1.78 | .07 | 6.30 ± 4.68 | 4.22 ± 3.72 | 1.71 | .07 | | 2.47 ± 6.33 | 2.01 ± 5.52 | 1.06 | .04 | 7.19 ± 3.95 | 5.72 ± 3.95 | 0.88 | .04 |
| ***mfcc5*** | 4.27 ± 6.61 | -3.77 ± 6.05 | 11.30^***^ | **.32** | 2.40 ± 5.97 | -4.37 ± 6.00 | 8.57^**^ | .26 | | -0.57 ± 6.6 | -8.12 ± 8.49 | 9.43^***^ | **.28** | 3.28 ± 6.44 | -3.13 ± 6.44 | 8.19^**^ | .25 |
| ***mfcc6*** | 5.80 ± 6.66 | 7.82 ± 6.46 | 2.60 | .10 | 4.27 ± 5.90 | 7.68 ± 5.73 | 4.28^*^ | .15 | | 2.23 ± 6.07 | 7.36 ± 6.81 | 5.2^**^ | .18 | 4.48 ± 5.65 | 7.50 ± 5.65 | 4.80^*^ | .17 |
| ***mfcc7*** | -0.53 ± 5.63 | -8.22 ± 4.84 | 14.54^***^ | **.38** | -0.58 ± 5.50 | -8.30 ± 4.82 | 14.01^***^ | **.37** | | -2.67 ±5.96 | -10.08 ± 4.72 | 12.37^***^ | **.34** | -0.14 ± 5.11 | -7.91 ± 5.11 | 16.76^***^ | **.41** |
| ***mfcc8*** | 3.94 ± 5.53 | 2.61 ± 4.67 | 0.42 | .02 | 2.90 ± 5.34 | 2.55 ± 4.29 | 0.03 | .00 | | 3.75 ± 6.46 | 2.19 ± 6.52 | 0.40 | .02 | 3.61 ± 5.50 | 2.50 ± 5.50 | 0.69 | .03 |
| ***mfcc9*** | 1.02 ± 3.97 | 3.54 ± 4.39 | 2.29 | .09 | 1.27 ± 5.07 | 3.14 ± 3.37 | 1.58 | .06 | | 1.23 ± 5.78 | 3.91 ± 4.10 | 1.81 | .07 | 1.41 ± 4.54 | 3.44 ± 4.54 | 2.56 | .10 |
| ***mfcc10*** | 4.25 ± 4.11 | 3.16 ± 3.71 | 1.89 | .07 | 3.76 ± 4.07 | 2.41 ± 3.61 | 1.12 | .04 | | 3.27 ± 4.28 | 0.08 ± 4.43 | 5.28^**^ | .18 | 4.17 ± 3.83 | 3.04 ± 3.83 | 0.66 | .03 |
| ***mfcc11*** | 1.20 ± 3.81 | 0.73 ± 3.60 | 2.65 | .10 | 1.40 ± 4.22 | 0.24 ± 3.49 | 2.05 | .08 | | -0.33 ±4.32 | -0.82 ± 4.30 | 0.70 | .03 | 0.98 ± 2.82 | 1.45 ± 2.82 | 2.67 | .10 |
| ***mfcc12*** | -2.92 ± 4.21 | -1.7 ± 2.85 | 0.70 | .03 | -2.39 ± 3.24 | -2.03 ± 3.54 | 0.07 | .00 | | -4.81 ±3.67 | -3.50 ± 2.58 | 0.97 | .04 | -3.21 ± 3.95 | -1.62 ± 3.95 | 1.69 | .07 |
| ***lsp0*** | 0.19 ± 0.04 | 0.20 ± 0.03 | 0.17 | .01 | 0.19 ± 0.02 | 0.19 ± 0.03 | 0.17 | .01 | | 0.19 ± 0.03 | 0.19 ± 0.02 | 0.01 | .00 | 0.20 ± 0.03 | 0.20 ± 0.03 | 0.05 | .00 |
| ***lsp1*** | 0.61 ± 0.06 | 0.61 ± 0.06 | 0.20 | .01 | 0.60 ± 0.05 | 0.60 ± 0.06 | 0.25 | .01 | | 0.55 ± 0.04 | 0.56 ± 0.05 | 0.20 | .01 | 0.62 ± 0.05 | 0.61 ± 0.05 | 0.10 | .00 |
| ***lsp2*** | 0.96 ± 0.06 | 0.97 ± 0.06 | 0.14 | .01 | 0.96 ± 0.06 | 0.96 ± 0.06 | 0.71 | .03 | | 0.91 ± 0.08 | 0.93 ± 0.06 | 1.04 | .04 | 0.97 ± 0.07 | 0.97 ± 0.07 | 0.01 | .00 |
| ***lsp3*** | 1.33 ± 0.07 | 1.29 ± 0.08 | 3.63^*^ | .13 | 1.33 ± 0.07 | 1.28 ± 0.08 | 3.95^*^ | .14 | | 1.28 ± 0.07 | 1.23 ± 0.08 | 5.51^**^ | .19 | 1.34 ± 0.07 | 1.28 ± 0.07 | 4.01^*^ | .14 |
| ***lsp4*** | 1.65 ± 0.08 | 1.59 ± 0.10 | 5.98^**^ | .20 | 1.65 ± 0.07 | 1.58 ± 0.10 | 6.11^**^ | .20 | | 1.61 ± 0.08 | 1.53 ± 0.10 | 10.54^***^ | **.31** | 1.66 ± 0.07 | 1.59 ± 0.07 | 6.15^**^ | .20 |
| ***lsp5*** | 2.00 ± 0.08 | 1.94 ± 0.11 | 6.43^**^ | .21 | 1.99 ± 0.07 | 1.92 ± 0.11 | 5.55^**^ | .19 | | 1.96 ± 0.07 | 1.87 ± 0.11 | 10.31^***^ | **.30** | 2.00 ± 0.06 | 1.94 ± 0.06 | 5.84^**^ | .20 |
| ***lsp6*** | 2.36 ± 0.08 | 2.30 ± 0.09 | 7.82^**^ | .25 | 2.37 ± 0.06 | 2.29 ± 0.10 | 7.60^**^ | .24 | | 2.34 ± 0.07 | 2.24 ± 0.11 | 13.97^***^ | **.37** | 2.37 ± 0.06 | 2.30 ± 0.06 | 8.27^**^ | .26 |
| ***lsp7*** | 2.72 ± 0.04 | 2.70 ± 0.04 | 3.31^*^ | .12 | 2.72 ± 0.04 | 2.69 ± 0.05 | 3.15 | .12 | | 2.71 ± 0.05 | 2.66 ± 0.06 | 7.37^**^ | .23 | 2.72 ± 0.04 | 2.69 ± 0.04 | 3.83^*^ | .14 |
| ***zcr*** | 0.03 ± 0.01 | 0.03 ± 0.01 | 0.77 | .03 | 0.03 ± 0.01 | 0.03 ± 0.01 | 2.38 | .09 | | 0.03 ± 0.01 | 0.03 ± 0.01 | 2.91 | .11 | 0.03 ± 0.01 | 0.03 ± 0.01 | 1.29 | .05 |
| ***vp*** | 0.53 ± 0.07 | 0.48 ± 0.04 | 4.01^*^ | .14 | 0.53 ± 0.06 | 0.49 ± 0.03 | 4.96^*^ | .17 | | 0.56 ± 0.05 | 0.52 ± 0.05 | 2.23 | .08 | 0.53 ± 0.07 | 0.49 ± 0.07 | 3.68^*^ | .13 |
| ***F0*** | 117.95±57.90 | 72.85 ± 32.10 | 6.23^**^ | .21 | 115.02±40.80 | 74.04 ± 24.28 | 9.51^***^ | .28 | | 123.7±41.30 | 87.16 ± 27.63 | 6.68^**^ | .22 | 117.66±50.16 | 76.0 ± 50.16 | 7.00^**^ | .23 |
| ***F0env*** | 293.63±47.04 | 265.81±52.47 | 4.67^*^ | .16 | 290.79±43.78 | 265.24±55.38 | 2.03 | .08 | | 257.83±46.59 | 220.76±52.57 | 6.33^**^ | .21 | 290.47±45.19 | 260.12±45.19 | 3.08 | .11 |

Note: ^*^, p < 0.05; ^**^, p < 0.01; ^***^, p < 0.001; In the column of η_p_^2^, we use bold for representing the features have large effect sizes.

**Table 2 Neutral emotion: the different acoustic features between depressed and healthy people under different tasks (male)**

|  | ***Video Watching*** | | | | ***Question Answering*** | | | | ***Text Reading*** | | | | ***Picture Describing*** | | | |
| --- | --- | --- | --- | --- | --- | --- | --- | --- | --- | --- | --- | --- | --- | --- | --- | --- |
|  | ***healthy*** | ***depressed*** | F | η_p_^2^ | ***healthy*** | ***depressed*** | F | η_p_^2^ | ***healthy*** | ***depressed*** | F | η_p_^2^ | ***healthy*** | ***depressed*** | F | η_p_^2^ |
| ***loudness*** | 0.42 ± 0.20 | 0.15 ± 0.15 | 15.85^***^ | **.40** | 0.42 ± 0.19 | 0.16 ± 0.17 | 16.11^***^ | **.40** | 0.52 ± 0.24 | 0.21 ± 0.22 | 13.06^***^ | **.36** | 0.37 ± 0.15 | 0.15 ± 0.15 | 21.41^***^ | **.47** |
| ***mfcc1*** | 1.73 ± 3.09 | 2.02 ± 3.35 | 0.05 | .00 | 1.79 ± 2.91 | 1.65 ± 3.01 | 0.25 | .01 | 2.48 ± 3.28 | 2.77 ± 2.10 | 0.24 | .01 | 0.98 ± 2.53 | 1.83 ± 2.88 | 0.73 | .03 |
| ***mfcc2*** | 9.07 ± 2.77 | 8.80 ± 2.47 | 0.27 | .01 | 8.92 ± 2.89 | 9.12 ± 2.56 | 0.09 | .00 | 8.84 ± 3.67 | 11.14 ± 4.09 | 2.25 | .09 | 9.02 ± 2.55 | 8.91 ± 2.37 | 0.03 | .00 |
| ***mfcc3*** | 9.06 ± 4.43 | 5.03 ± 3.46 | 6.13^**^ | .20 | 10.37 ± 4.29 | 4.71 ± 3.64 | 13.74^***^ | **.36** | 11.15 ± 5.12 | 5.30 ± 4.80 | 9.67^***^ | **.29** | 9.03 ± 3.72 | 4.65 ± 3.31 | 9.23^***^ | **.28** |
| ***mfcc4*** | 6.87 ± 4.35 | 5.00 ± 4.21 | 1.15 | .05 | 6.28 ± 4.40 | 4.74 ± 3.84 | 0.88 | .04 | 1.96 ± 6.13 | 1.38 ± 5.33 | 0.65 | .03 | 7.16 ± 3.50 | 5.22 ± 3.09 | 2.71 | .10 |
| ***mfcc5*** | 3.01 ± 6.77 | -3.97 ± 6.05 | 7.05^**^ | .23 | 1.96 ± 6.37 | -5.19 ± 6.76 | 7.63^**^ | .24 | -3.45 ± 6.77 | -10.91 ± 7.51 | 10.27^***^ | .30 | 3.62 ± 5.96 | -3.00 ± 4.31 | 10.20^***^ | **.30** |
| ***mfcc6*** | 5.29 ± 6.75 | 8.32 ± 5.36 | 4.45^*^ | .16 | 4.34 ± 6.05 | 7.15 ± 5.97 | 4.13^**^ | .15 | 1.90 ± 6.48 | 7.92 ± 6.50 | 7.28^**^ | .24 | 5.20 ± 4.81 | 7.04 ± 5.10 | 7.18^**^ | .23 |
| ***mfcc7*** | -1.15 ± 6.28 | -7.55 ± 5.60 | 7.54^**^ | .24 | -1.43 ± 5.72 | -8.80 ± 5.50 | 11.12^***^ | **.32** | -3.54 ± 6.31 | -10.34 ± 4.98 | 8.81^**^ | .27 | -0.64 ± 4.31 | -7.64 ± 4.58 | 17.90^***^ | **.43** |
| ***mfcc8*** | 3.10 ± 5.89 | 2.98 ± 4.66 | 0.01 | .00 | 2.85 ± 5.47 | 2.41 ± 4.55 | 0.13 | .01 | 0.88 ± 5.85 | 0.36 ± 6.24 | 0.04 | .00 | 3.44 ± 3.92 | 2.40 ± 3.48 | 1.09 | .04 |
| ***mfcc9*** | 0.90 ± 4.90 | 4.02 ± 4.41 | 2.69 | .10 | 0.63 ± 5.49 | 3.13 ± 3.57 | 1.73 | .07 | -1.90 ± 5.56 | 1.18 ± 4.04 | 2.54 | .10 | 1.93 ± 2.82 | 3.13 ± 2.24 | 3.77^*^ | .14 |
| ***mfcc10*** | 4.54 ± 4.37 | 3.36 ± 3.83 | 0.67 | .03 | 4.05 ± 3.59 | 2.05 ± 3.42 | 1.97 | .08 | 2.51 ± 4.67 | 0.37 ± 4.61 | 2.81 | .11 | 4.22 ± 3.02 | 2.75 ± 3.02 | 1.45 | .06 |
| ***mfcc11*** | 1.72 ± 3.70 | 1.06 ± 3.86 | 1.25 | .05 | 1.57 ± 4.25 | 0.60 ± 3.28 | 2.83 | .11 | 2.23 ± 4.05 | 1.22 ± 4.58 | 0.59 | .02 | 0.91 ± 2.36 | 1.59 ± 2.86 | 3.00 | .11 |
| ***mfcc12*** | -3.29 ± 3.81 | -1.64 ± 3.08 | 1.37 | .05 | -2.70 ± 3.81 | -2.04 ± 3.38 | 0.25 | .01 | -5.06 ± 4.33 | -3.48 ± 2.14 | 1.59 | .06 | -2.74 ± 2.77 | -1.38 ± 2.24 | 1.71 | .07 |
| ***lsp0*** | 0.18 ± 0.02 | 0.19 ± 0.04 | 0.32 | .01 | 0.19 ± 0.02 | 0.19 ± 0.03 | 0.60 | .02 | 0.19 ± 0.03 | 0.19 ± 0.02 | 0.14 | .01 | 0.19 ± 0.03 | 0.19 ± 0.03 | 0.05 | .00 |
| ***lsp1*** | 0.60 ± 0.05 | 0.60 ± 0.06 | 0.13 | .01 | 0.60 ± 0.05 | 0.59 ± 0.06 | 0.26 | .01 | 0.55 ± 0.05 | 0.56 ± 0.04 | 0.42 | .02 | 0.62 ± 0.05 | 0.60 ± 0.05 | 0.67 | .03 |
| ***lsp2*** | 0.96 ± 0.07 | 0.97 ± 0.07 | 0.36 | .01 | 0.95 ± 0.07 | 0.96 ± 0.06 | 0.07 | .00 | 0.90 ± 0.08 | 0.94 ± 0.06 | 1.82 | .07 | 0.98 ± 0.05 | 0.97 ± 0.05 | 0.36 | .01 |
| ***lsp3*** | 1.33 ± 0.07 | 1.29 ± 0.09 | 2.31 | .09 | 1.33 ± 0.07 | 1.27 ± 0.08 | 4.50^**^ | .16 | 1.26 ± 0.07 | 1.22 ± 0.08 | 3.57^*^ | .13 | 1.35 ± 0.06 | 1.28 ± 0.07 | 6.55^**^ | .21 |
| ***lsp4*** | 1.65 ± 0.08 | 1.59 ± 0.10 | 4.14^*^ | .15 | 1.65 ± 0.07 | 1.57 ± 0.10 | 6.32^**^ | .21 | 1.59 ± 0.08 | 1.51 ± 0.10 | 8.88^**^ | .27 | 1.67 ± 0.07 | 1.59 ± 0.08 | 8.53^**^ | .26 |
| ***lsp5*** | 2.00 ± 0.07 | 1.94 ± 0.10 | 4.94^*^ | .17 | 1.99 ± 0.07 | 1.91 ± 0.11 | 7.05^**^ | .23 | 1.95 ± 0.07 | 1.87 ± 0.10 | 9.27^***^ | **.28** | 2.01 ± 0.06 | 1.94 ± 0.09 | 8.45^**^ | .26 |
| ***lsp6*** | 2.37 ± 0.06 | 2.30 ± 0.10 | 6.90^**^ | .22 | 2.36 ± 0.06 | 2.28 ± 0.10 | 9.18^***^ | **.28** | 2.33 ± 0.07 | 2.23 ± 0.10 | 12.71^***^ | **.35** | 2.38 ± 0.06 | 2.30 ± 0.09 | 9.23^***^ | **.28** |
| ***lsp7*** | 2.72 ± 0.04 | 2.70 ± 0.04 | 2.62 | .10 | 2.72 ± 0.04 | 2.69 ± 0.05 | 3.68^*^ | .13 | 2.70 ± 0.05 | 2.65 ± 0.06 | 6.54^**^ | .22 | 2.73 ± 0.03 | 2.70 ± 0.04 | 4.55^*^ | .16 |
| ***zcr*** | 0.02 ± 0.01 | 0.03 ± 0.01 | 2.26 | .09 | 0.03 ± 0.01 | 0.03 ± 0.01 | 4.38^*^ | .15 | 0.03 ± 0.01 | 0.04 ± 0.01 | 3.95^*^ | .14 | 0.03 ± 0.01 | 0.03 ± 0.01 | 0.74 | .03 |
| ***vp*** | 0.54 ± 0.07 | 0.49 ± 0.04 | 4.87^*^ | .17 | 0.54 ± 0.06 | 0.49 ± 0.03 | 4.88^*^ | .17 | 0.57 ± 0.05 | 0.53 ± 0.04 | 2.74 | .10 | 0.53 ± 0.05 | 0.49 ± 0.03 | 6.53^**^ | .21 |
| ***F0*** | 122.82±51.63 | 74.09±25.93 | 8.76^**^ | .27 | 115.29±42.06 | 76.56 ± 22.97 | 9.72^***^ | **.29** | 128.62±37.78 | 91.02 ± 29.72 | 8.72^**^ | .27 | 118.96 ± 42.34 | 71.86 ± 26.93 | 11.05^***^ | **.32** |
| ***F0env*** | 286.67±46.94 | 262.62±50.95 | 1.81 | .07 | 282.28±46.18 | 255.22±56.26 | 1.98 | .08 | 258.62±48.11 | 218.33 ± 49.58 | 7.98^**^ | .25 | 298.75 ± 35.9 | 266.38 ± 41.58 | 4.41^*^ | .16 |

Note: ^*^, p < 0.05; ^**^, p < 0.01; ^***^, p < 0.001; In the column of η_p_^2^, we use bold for representing the features have large effect sizes.

**Table 3 Negative emotion: the different acoustic features between depressed and healthy people under different tasks (male)**

|  | ***Video Watching*** | | | | ***Question Answering*** | | | | ***Text Reading*** | | | | ***Picture Describing*** | | | |
| --- | --- | --- | --- | --- | --- | --- | --- | --- | --- | --- | --- | --- | --- | --- | --- | --- |
|  | ***healthy*** | ***depressed*** | F | η_p_^2^ | ***healthy*** | ***depressed*** | F | η_p_^2^ | ***healthy*** | ***depressed*** | F | η_p_^2^ | ***healthy*** | ***depressed*** | F | η_p_^2^ |
| ***loudness*** | 0.39 ± 0.16 | 0.15 ± 0.15 | 18.36^***^ | **.43** | 0.38 ± 0.17 | 0.14 ± 0.14 | 18.33^***^ | **.43** | 0.51 ± 0.25 | 0.20 ± 0.20 | 12.39^***^ | **.34** | 0.37 ± 0.16 | 0.15 ± 0.15 | 17.75^***^ | **.42** |
| ***mfcc1*** | 0.82 ± 3.50 | 1.36 ± 3.31 | 0.23 | .01 | 0.78 ± 2.8 | 1.90 ± 3.21 | 0.86 | .03 | 2.61 ± 2.97 | 2.35 ± 2.31 | 0.24 | .01 | 1.22 ± 3.23 | 1.87 ± 3.34 | 0.48 | .02 |
| ***mfcc2*** | 8.73 ± 3.20 | 9.51 ± 2.48 | 0.53 | .02 | 8.75 ± 3.09 | 8.83 ± 2.66 | 0.01 | .00 | 8.96 ± 3.94 | 10.65 ± 4.01 | 1.29 | .05 | 8.76 ± 2.78 | 8.73 ± 2.60 | 0.01 | .00 |
| ***mfcc3*** | 9.02 ± 4.56 | 3.70 ± 4.04 | 9.89^***^ | **.29** | 9.23 ± 3.97 | 4.18 ± 3.49 | 11.26^***^ | **.32** | 10.89 ± 4.84 | 4.71 ± 4.72 | 11.66^***^ | **.33** | 8.60 ± 4.30 | 4.35 ± 3.52 | 6.96^**^ | .22 |
| ***mfcc4*** | 7.37 ± 4.75 | 5.38 ± 4.00 | 1.23 | .05 | 6.94 ± 4.15 | 4.36 ± 4.18 | 2.62 | .10 | 3.21 ± 5.97 | 2.08 ± 5.23 | 1.22 | .05 | 6.91 ± 4.00 | 5.28 ± 4.00 | 1.32 | .05 |
| ***mfcc5*** | 3.69 ± 6.12 | -3.92 ± 6.27 | 9.70^***^ | **.29** | 3.42 ± 6.61 | -4.12 ± 6.18 | 9.96^***^ | **.29** | -1.46 ± 6.78 | -9.51 ± 7.29 | 12.95^***^ | **.35** | 3.48 ± 5.92 | -3.01 ± 4.89 | 8.92^**^ | .27 |
| ***mfcc6*** | 4.96 ± 6.81 | 7.32 ± 5.47 | 3.53 | .13 | 5.40 ± 6.18 | 7.90 ± 5.71 | 3.25^*^ | .12 | 2.72 ± 6.25 | 7.78 ± 6.65 | 5.39^**^ | .18 | 5.60 ± 5.93 | 8.09 ± 6.44 | 4.27^*^ | .15 |
| ***mfcc7*** | -0.41 ± 5.91 | -7.83 ± 5.44 | 10.50^***^ | **.30** | -0.45 ± 5.42 | -7.77 ± 5.08 | 12.08^***^ | **.33** | -2.24 ± 5.67 | -10.59 ± 5.58 | 15.31^***^ | **.39** | -0.10 ± 5.06 | -7.30 ± 4.36 | 14.38^***^ | **.37** |
| ***mfcc8*** | 3.67 ± 6.06 | 2.38 ± 4.16 | 0.36 | .01 | 2.87 ± 4.94 | 2.73 ± 4.63 | 0.11 | .00 | 1.36 ± 6.87 | 0.85 ± 6.19 | 0.12 | .00 | 4.03 ± 4.63 | 2.52 ± 4.13 | 1.08 | .04 |
| ***mfcc9*** | 1.17 ± 5.15 | 3.64 ± 4.02 | 1.66 | .06 | 1.47 ± 5.37 | 3.42 ± 3.17 | 1.09 | .04 | -0.96 ± 5.97 | 1.91 ± 4.5 | 1.82 | .07 | 2.06 ± 4.14 | 3.79 ± 3.21 | 2.11 | .08 |
| ***mfcc10*** | 4.56 ± 3.98 | 3.14 ± 4.11 | 1.29 | .05 | 4.68 ± 3.38 | 2.05 ± 3.71 | 3.43^**^ | .13 | 2.63 ± 4.75 | 0.33 ± 4.17 | 4.45^*^ | .16 | 4.47 ± 3.48 | 3.20 ± 3.88 | 0.75 | .03 |
| ***mfcc11*** | 0.34 ± 3.57 | 0.46 ± 3.28 | 2.14 | .08 | 1.10 ± 3.63 | 0.40 ± 3.53 | 1.85 | .07 | 0.49 ± 4.41 | 0.22 ± 4.64 | 0.24 | .01 | 1.12 ± 2.91 | 1.63 ± 3.48 | 1.34 | .05 |
| ***mfcc12*** | -2.71 ± 3.47 | -2.27 ± 3.81 | 0.12 | .01 | -2.43 ± 3.28 | -1.46 ± 3.28 | 0.82 | .03 | -4.67 ± 3.68 | -4.01 ± 2.75 | 0.25 | .01 | -2.86 ± 3.32 | -1.39 ± 2.52 | 1.49 | .06 |
| ***lsp0*** | 0.19 ± 0.03 | 0.20 ± 0.03 | 0.13 | .01 | 0.19 ± 0.03 | 0.20 ± 0.03 | 0.01 | .00 | 0.19 ± 0.03 | 0.20 ± 0.02 | 0.46 | .02 | 0.19 ± 0.03 | 0.19 ± 0.03 | 0.11 | .00 |
| ***lsp1*** | 0.62 ± 0.05 | 0.62 ± 0.06 | 0.10 | .00 | 0.61 ± 0.05 | 0.60 ± 0.07 | 0.27 | .01 | 0.56 ± 0.05 | 0.57 ± 0.03 | 0.38 | .02 | 0.62 ± 0.06 | 0.61 ± 0.06 | 0.26 | .01 |
| ***lsp2*** | 0.96 ± 0.06 | 0.98 ± 0.06 | 0.61 | .02 | 0.97 ± 0.06 | 0.97 ± 0.07 | 0.18 | .01 | 0.91 ± 0.07 | 0.94 ± 0.06 | 1.60 | .06 | 0.98 ± 0.07 | 0.97 ± 0.06 | 0.21 | .01 |
| ***lsp3*** | 1.33 ± 0.07 | 1.29 ± 0.08 | 2.66 | .10 | 1.34 ± 0.07 | 1.28 ± 0.09 | 4.40^*^ | .15 | 1.28 ± 0.08 | 1.23 ± 0.08 | 5.23^**^ | .18 | 1.35 ± 0.07 | 1.29 ± 0.08 | 5.13^**^ | .18 |
| ***lsp4*** | 1.65 ± 0.08 | 1.60 ± 0.10 | 5.21^**^ | .18 | 1.66 ± 0.07 | 1.59 ± 0.10 | 6.83^**^ | .22 | 1.60 ± 0.09 | 1.52 ± 0.10 | 9.30^***^ | **.28** | 1.67 ± 0.07 | 1.59 ± 0.09 | 6.77^**^ | .22 |
| ***lsp5*** | 2.00 ± 0.07 | 1.93 ± 0.10 | 6.24^**^ | .21 | 2.00 ± 0.06 | 1.93 ± 0.11 | 6.44^**^ | .21 | 1.95 ± 0.08 | 1.87 ± 0.10 | 10.01^***^ | **.29** | 2.01 ± 0.06 | 1.94 ± 0.09 | 6.64^**^ | .22 |
| ***lsp6*** | 2.36 ± 0.07 | 2.29 ± 0.10 | 7.98^**^ | .25 | 2.37 ± 0.06 | 2.30 ± 0.10 | 8.11^**^ | .25 | 2.34 ± 0.07 | 2.23 ± 0.11 | 14.67^***^ | **.38** | 2.38 ± 0.06 | 2.30 ± 0.09 | 8.78^**^ | .27 |
| ***lsp7*** | 2.72 ± 0.04 | 2.69 ± 0.06 | 3.69^*^ | .13 | 2.72 ± 0.04 | 2.70 ± 0.05 | 3.06 | .11 | 2.70 ± 0.05 | 2.65 ± 0.06 | 7.73^**^ | .24 | 2.73 ± 0.04 | 2.70 ± 0.05 | 3.87^*^ | .14 |
| ***zcr*** | 0.03 ± 0.01 | 0.03 ± 0.01 | 1.60 | .06 | 0.03 ± 0.01 | 0.03 ± 0.01 | 1.53 | .06 | 0.03 ± 0.01 | 0.04 ± 0.01 | 6.07^**^ | .20 | 0.03 ± 0.01 | 0.03 ± 0.01 | 0.58 | .02 |
| ***vp*** | 0.53 ± 0.06 | 0.49 ± 0.04 | 3.75^*^ | .14 | 0.52 ± 0.05 | 0.48 ± 0.03 | 4.52^*^ | .16 | 0.56 ± 0.05 | 0.53 ± 0.04 | 2.92 | .11 | 0.53 ± 0.06 | 0.48 ± 0.03 | 4.44^*^ | .16 |
| ***F0*** | 116.33± 50.03 | 75.93 ± 30.20 | 6.81^**^ | .22 | 113.15 ± 42.94 | 71.31 ± 20.53 | 8.95^***^ | **.27** | 133.14 ± 42.05 | 90.75 ± 28.54 | 8.93^**^ | .27 | 114.91 ± 45.92 | 71.33 ± 25.78 | 9.08^***^ | **.27** |
| ***F0env*** | 288.83±43.53 | 269.05± 56.02 | 1.62 | .06 | 292.91 ± 42.87 | 267.54 ± 54.49 | 3.12 | .11 | 263.17 ± 47.26 | 223.62 ± 50.04 | 6.79^**^ | .22 | 298.75 ± 44.82 | 266.86 ± 49.57 | 2.94 | .11 |

Note: ^*^, p < 0.05; ^**^, p < 0.01; ^***^, p < 0.001; In the column of η_p_^2^, we use bold for representing the features have large effect sizes.
