# Supplementary material for: Acoustic differences between healthy and depressed people: a cross-situation study
Source: BMC Psychiatry. 2019 Oct 15;19:300. doi: 10.1186/s12888-019-2300-7 (PMC6794822; doi:10.1186/s12888-019-2300-7)
Supplement: Supplementary file 3 — Additional file 3. Stimuli in the tasks. [file 12888_2019_2300_MOESM3_ESM.docx]

**Materials**

To evaluate the validities of the materials used for emotional priming, a pilot testing had been implemented. A group of 30 healthy people rated valence and arousal of each stimuli using a 7 Likert scale. In terms of valence, 1 represents the happiest and 7 represents the unhappiest. In terms of arousal, 1 represents the lowest and 7 represents the highest.

The ratings of pictures were regarded as ground truth to the other three types of materials, because they were chosen from a validated and commonly-used database (Lu, Hui, & Yu-Xia, 2005). Table 1 shows the mean ratings of all materials. Similar to pictures’ ratings, the ratings of other three types of materials indicate that they are able to induce corresponding emotions. The results supported that these materials are effective.

**Table 1 Ratings of materials**

|  | **Positive (happy)** | | **Neutral** | | **Negative (sadness)** | |
| --- | --- | --- | --- | --- | --- | --- |
|  | *valence* | *arousal* | *valence* | *arousal* | *valence* | *arousal* |
| ***Video*** | 5.5 | 5.1 | 4.3 | 3.7 | 2.3 | 4.5 |
| ***Audio***^1^ | 6.1 | 5.0 | 3.7 | 4.2 | 2.2 | 4.9 |
|  | 5.9 | 4.7 | 4.1 | 3.8 | 2.6 | 4.7 |
|  | 5.6 | 5.3 | 4.3 | 3.4 | 2.6 | 4.6 |
| ***Text*** | 5.8 | 3.6 | 4.2 | 2.8 | 3.0 | 3.5 |
| ***Picture***^2^ | 5.2 | 4.4 | 3.7 | 3.4 | 2.9 | 4.5 |
|  | 5.9 | 4.8 | 4.2 | 3.1 | 2.0 | 4.9 |

^1^ there are three audio materials used for each emotion in task Question Answering

^2^ there are two pictures used for each emotion in task Picture Describing

**Video**

Three videos were selected in task Video Watching. Positive video stimulus was derived from cartoon *Larva* season 1 episode 58. A clip from documentary *Space Millennium* was used for neutral emotional priming. The video for negative stimulus was edited from a Chinese film *The Stand-In*. The durations of them are approximately 1 minute and 30 seconds.

**Audios**

There were nine audio questions for the task Question Answering. Each emotional valence had 3 audio questions. One instance of positive question is “please share us with your happiest memory then describe its details.”. one example of negative question is “Is there anything that makes you feel remorseful or self-accusation?”. Neutral question likes “How do you think of yourself?”. To eliminate the impact of experimenters’ voices on participants, all 9 audio questions were read out by one female experimenter, and these speeches were recorded. The recording of these questions were automatically broadcasted during the experiment.

**Texts**

The task Text Reading consisted of three texts. The positive text was a paragraph about the Chinese Spring Festival. The negative one was a paragraph excerpted from *Les Miserables*. The neutral one was a paragraph which objectively describes a famous Chinese bridge called *Lugou* bridge. Each text had about 150 Chinese characters.

**Pictures**

The task Picture Describing had six pictures, which were chosen from the Chinese emotion pictures database (Lu, Hui, & Yu-Xia, 2005). Each emotional valence had two pictures. Half of them were face expressions, and the others were scene pictures. There was one sentence “Does this picture remind you of something?” under every picture to remind participants to imagine.
