# Supplementary material for: Acoustic differences between healthy and depressed people: a cross-situation study
Source: BMC Psychiatry. 2019 Oct 15;19:300. doi: 10.1186/s12888-019-2300-7 (PMC6794822; doi:10.1186/s12888-019-2300-7)
Supplement: Supplementary file 4 — Additional file 4. Box-whisker plots of loudness, MFCC5, and MFCC7 in each emotion. [file 12888_2019_2300_MOESM4_ESM.docx]

**Positive emotion**

**Loudness**


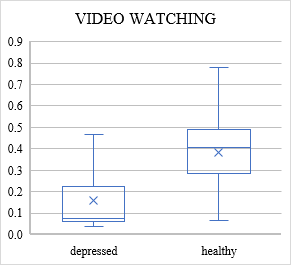

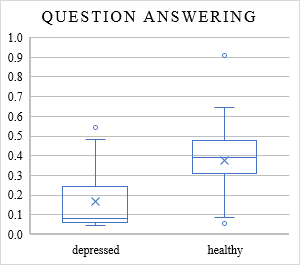


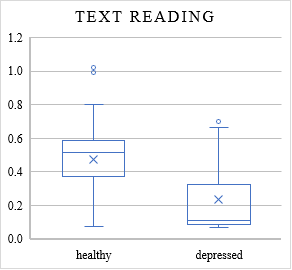

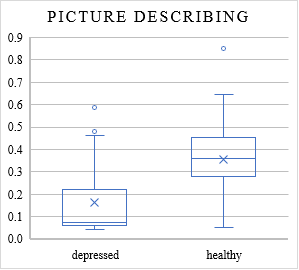


“×” represents mean

circle represents outlier

**Positive emotion**

**MFCC5**


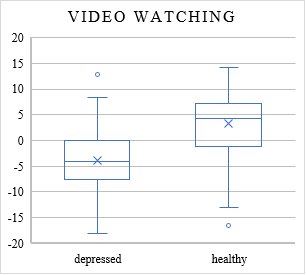

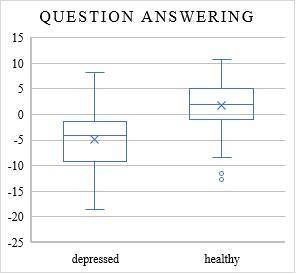


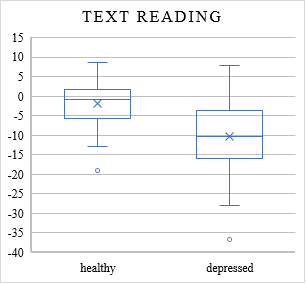

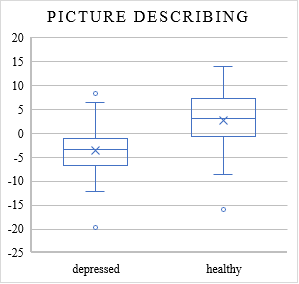


“×” represents mean

circle represents outlier

**Positive emotion**

**MFCC7**


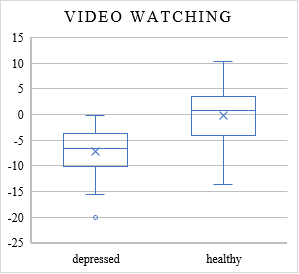

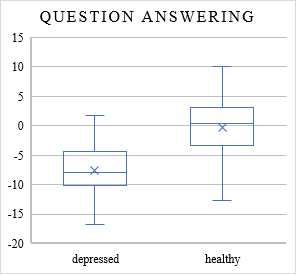


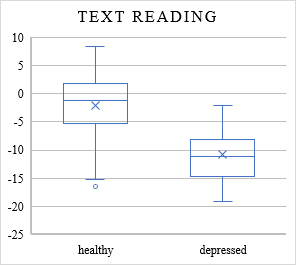

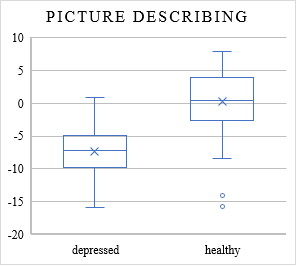


“×” represents mean

circle represents outlier

**Neutral emotion**

**Loudness**


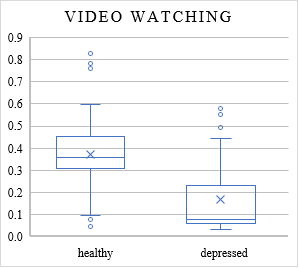

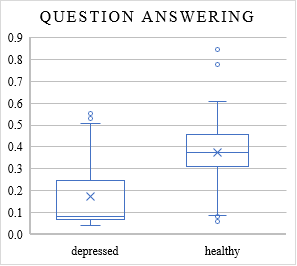


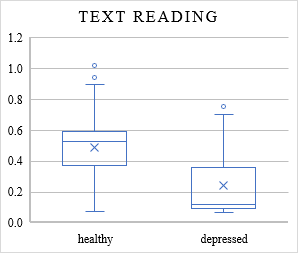

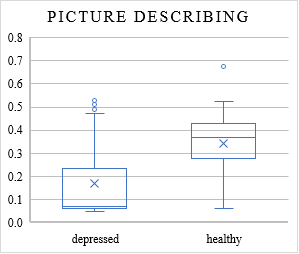


“×” represents mean

circle represents outlier

**Neutral emotion**

**MFCC5**


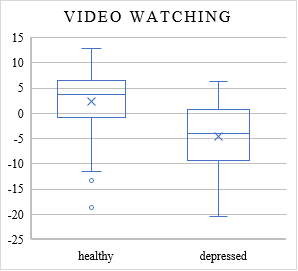

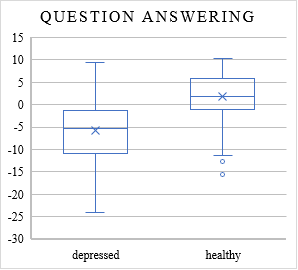


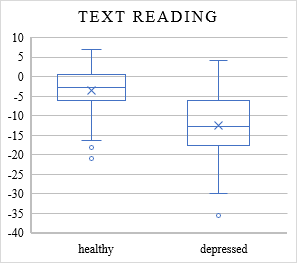

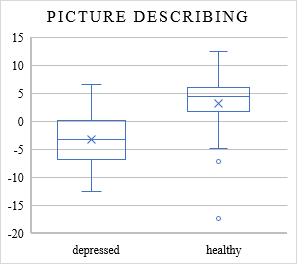


“×” represents mean

circle represents outlier

**Neutral emotion**

**MFCC7**


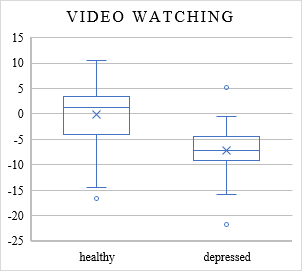

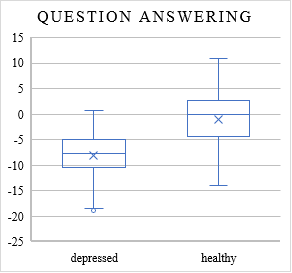


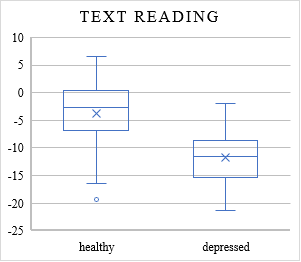

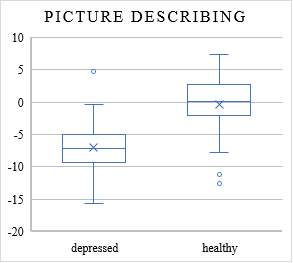


“×” represents mean

circle represents outlier

**Negative emotion**

**Loudness**


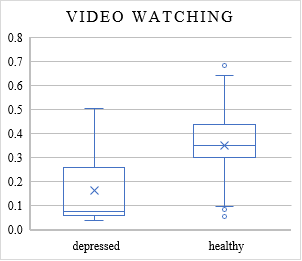

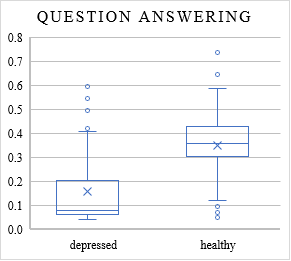


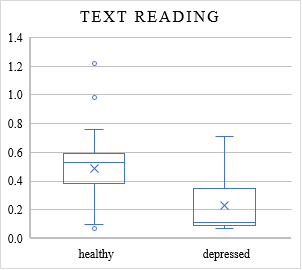

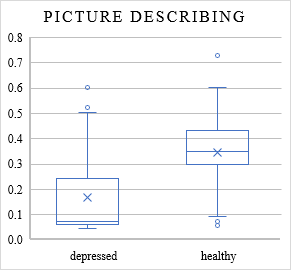


“×” represents mean

circle represents outlier

**Negative emotion**

**MFCC5**


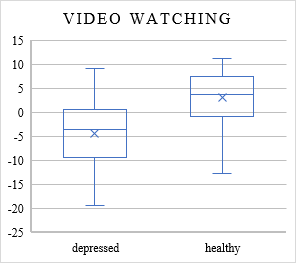

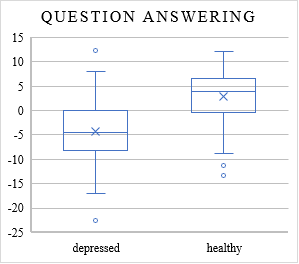


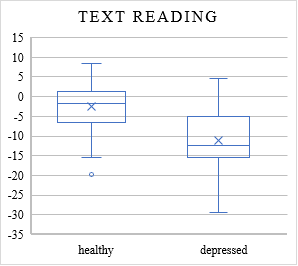

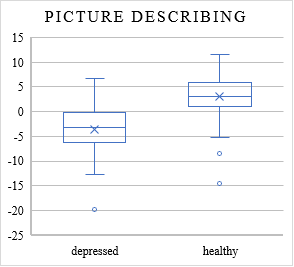


“×” represents mean

circle represents outlier

**Negative emotion**

**MFCC7**


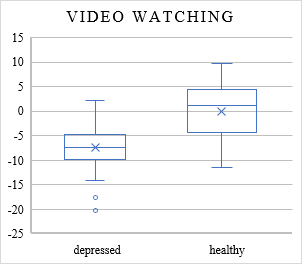

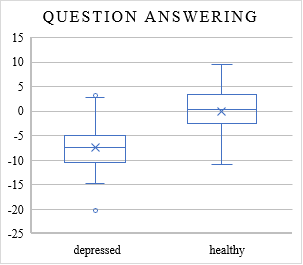


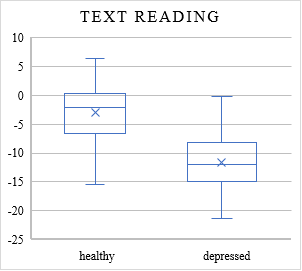

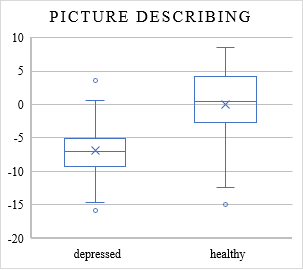


“×” represents mean

circle represents outlier
